# Deciphering the Molecular Basis of Wine Yeast Fermentation Traits Using a Combined Genetic and Genomic Approach

**DOI:** 10.1534/g3.111.000422

**Published:** 2011-09-01

**Authors:** Chloé Ambroset, Maud Petit, Christian Brion, Isabelle Sanchez, Pierre Delobel, Cyprien Guérin, Hélène Chiapello, Pierre Nicolas, Frédéric Bigey, Sylvie Dequin, Bruno Blondin

**Affiliations:** * Institut National de la Recherche Agronomique (INRA), UMR1083, and; ‡ Montpellier SupAgro, UMR 1083 Sciences pour l'Oenologie, F-34060 Montpellier, and; † INRA, UR1077 Mathématique Informatique et Génome, F-78352 Jouy-en-Josas, France

**Keywords:** wine yeast, fermentation, QTL, transcriptome, p-aminobenzoate

## Abstract

The genetic basis of the phenotypic diversity of yeast is still poorly understood. Wine yeast strains have specific abilities to grow and ferment under stressful conditions compared with other strains, but the genetic basis underlying these traits is unknown. Understanding how sequence variation influences such phenotypes is a major challenge to address adaptation mechanisms of wine yeast. We aimed to identify the genetic basis of fermentation traits and gain insight into their relationships with variations in gene expression among yeast strains. We combined fermentation trait QTL mapping and expression profiling of fermenting cells in a segregating population from a cross between a wine yeast derivative and a laboratory strain. We report the identification of QTL for various fermentation traits (fermentation rates, nitrogen utilization, metabolites production) as well as expression QTL (eQTL). We found that many transcripts mapped to several eQTL hotspots and that two of them overlapped with QTL for fermentation traits. A QTL controlling the maximal fermentation rate and nitrogen utilization overlapping with an eQTL hotspot was dissected. We functionally demonstrated that an allele of the *ABZ1* gene, localized in the hotspot and involved in *p*-aminobenzoate biosynthesis, controls the fermentation rate through modulation of nitrogen utilization. Our data suggest that the laboratory strain harbors a defective *ABZ1* allele, which triggers strong metabolic and physiological alterations responsible for the generation of the eQTL hotspot. They also suggest that a number of gene expression differences result from some alleles that trigger major physiological disturbances.

Industrial wine yeast strains exhibit specific traits making them suitable for alcoholic fermentation. These yeasts are specifically adapted to stressful conditions and can ferment efficiently under conditions of high ethanol concentrations, low pH, nutrient starvation, while producing suitable metabolites, especially aroma compounds. Although a large phenotypic diversity has been described, the molecular basis remains largely unknown. Various molecular mechanisms may contribute to wine yeast adaptation, including gene deletions or amplifications, chromosome polyploidy (Dunham *et al.* 2002; Dunn *et al.* 2005; Infante *et al.* 2003; Perez-Ortin *et al.* 2002; Rachidi *et al.* 1999), and nucleotide polymorphisms (Borneman *et al.* 2008; Doniger *et al.* 2008; Goffeau *et al.* 1996; Kellis *et al.* 2003; Liti *et al.* 2009; Schacherer *et al.* 2009; Wei *et al.* 2007). Analysis of the complete genome sequence of a commercial wine yeast also revealed that new (non-*Saccharomyces*) genes arising from horizontal gene transfer were present in the wine strains (Novo *et al.* 2009).

Linking genetic variation with phenotypic diversity will help to increase our understanding of the adaptation of yeast to industrial stressful environments and will facilitate strain improvement through breeding and genetic engineering. Quantitative trait locus (QTL) mapping is a proven approach to map the genetic variation responsible for quantitative traits in *S. cerevisiae*. It was successfully applied to high-temperature growth (Sinha *et al.* 2008; Sinha *et al.* 2006; Steinmetz *et al.* 2002), sporulation (Ben-Ari *et al.* 2006; Deutschbauer and Davis 2005; Gerke *et al.* 2006; Katou *et al.* 2009), cell morphology (Nogami *et al.* 2007), drug sensitivity (Kim and Fay, 2007), ethanol tolerance and growth (Hu *et al.* 2007; Katou *et al.* 2009; Smith and Kruglyak 2008), and flocculation (Brauer *et al.* 2006). QTL approaches have also been used to dissect the molecular basis of several wine yeast metabolic traits such as acetic acid production, hydrogen sulphide production, and release of volatile phenol (Marullo *et al.* 2006).

Several studies have highlighted the role of expression variations on associated phenotypes in yeast (Brown *et al.* 2008; Cavalieri *et al.* 2000; Fay *et al.* 2004). Variations in expression between wine strains have been reported in various genome-wide analyses (Rossignol 2004; Zuzuarregui *et al.* 2006). However, their impact on strain properties is poorly understood. Genomic approaches to variations in gene expression have been used to establish relationships between differences in transcript abundance and genetic polymorphisms via the mapping of expression QTL (eQTL) (Jansen and Nap 2001; Brem *et al.* 2002; Yvert *et al.* 2003; Brem and Kruglyak 2005; Ronald and Akey 2007; Ansel *et al.* 2008)). Combining the search of eQTL with that of QTL of industrially relevant traits may be of interest to gain insight into the relationships between expression variations and wine yeast traits. Indeed when an eQTL colocalizes with a phenotypic QTL, one can hypothesize that a common polymorphism controls transcription differences and the physiological trait.

Here, we report an integrated approach where we searched for both phenotypic fermentation QTL and eQTL in the laboratory yeast strain S288c and a haploid derivative of the EC1118 industrial wine strain. We identified QTL for various fermentation traits, including kinetic traits, as well as eQTL for many transcripts. We show that eQTL display hotspot regions that control many transcripts and interestingly, two of them overlap with fermentation traits QTL. We functionally characterized a QTL that controlled the maximal fermentation rate and showed that a gene involved in *p*-aminobenzoate synthesis (*ABZ1*), probably defective in the strain S288C, modulates the fermentation rate by controlling nitrogen utilization. The relationships between structural alteration of *ABZ1* and eQTL hotspot generation are discussed.

## Materials and Methods

### Strains, growth conditions, and fermentation conditions

**Figure 1:**
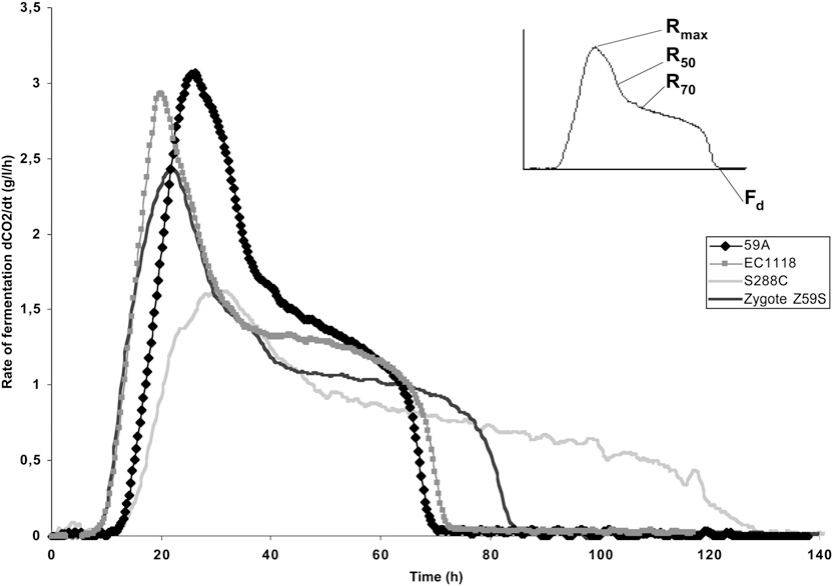
Fermentation profiles of industrial and laboratory strains. Fermentation rate profiles of the industrial wine yeast EC1118 (gray squares), its haploid derivative 59A (black diamonds), the laboratory strain S288C (gray line), and the hybrid Z59S (black line).

The two parental strains compared in this study are the standard S288c (*MAT*α; *SUC2; gal2*) strain and a haploid derivative of the industrial EC1118 (HO/ho) wine yeast strain, which is referred to as 59A (*MAT*a; ho). This strain is phototrophic and has fermentation properties close to the diploid EC1118 strain (see Figure 1). The strains BY4742 (MATα; his3Δ1; leu2Δ0; lys2Δ0; ura3Δ0 and BY4742∆*ABZ1* (Matα; his3Δ1; leu2Δ0; lys2Δ0; ura3Δ0; YNR033w::kanMX4) were used for hemizygous constructions.

Fermentation experiments and precultures were carried out at 28°C on synthetic MS300 medium, which mimics a natural must described in Bely *et al.* (1990). After preculture in 125 ml shake flasks for 24 h, fermentations were performed in 1.2 l fermenters equipped with airlocks to maintain anaerobiosis and with constant stirring.

### Construction of strains for functional analysis

Hybrid strains BY4742∆*ABZ1*/59A and BY4742/59A∆*ABZ1* were constructed for the functional characterization of the *ABZ1* candidate gene via a reciprocal hemizygosity analysis.

The BY4742∆*ABZ1*strain, which isogenic to S288c but with a copy of the *ABZ1* gene deleted, was obtained from the Euroscarff society. The copy of *ABZ1* in the 59A strain was deleted and replaced with a *KanMX4* deletion module using the short-flanking homology PCR (SFH-PCR) technique (Schiestl and Gietz 1989). The two custom 60-mer primers used begin with 40 nucleotides that are identical to the upstream or the downstream region of *ABZ1* followed by 20 nucleotides that can amplify the *KanMX4* module, *ABZ1* forward: ATGCTGTCCGATACAATTGACACAAAGCAACAACAGCAACTTCGTACGCTGCAGGTCGAC; and *ABZ1* reverse: CTACATGAAAATTTGCAAGTTGCTCTCCAACTTGGTGTACGCATAGGCCACTAGTGGATCTG. After transformation using the lithium acetate transformation protocol of Schiestl and Gietz (1989), yeasts were grown with YEPD plus geneticin (200 µg/l) overnight at 28°C to select cells with the *KanMX4* integrated module. Clones were finally tested for integration by PCR on genomic DNA with the primers ABZ1 forwardctrl: TTATCGGTGCGGCAATAAAG and ABZ1 reversectrl: TATGTGACCGTCAGGACG.

### Measure of phenotypic parameters

To characterize the fermentation parameters, the fermenters were automatically weighed every 20 min and the rate of CO_2_ production was calculated from the weight loss by a method of polynomial smoothing. The number of cells was determined with an electronic particle counter (Beckman Coulter). At the end of fermentation, samples were analyzed by high-pressure liquid chromatography (HPLC) using an HPX-87H ion-exclusion column (Bio-Rad) to assess the sugars (glucose, fructose), the organic acids (succinic acid, acetic acid, pyruvic acid), and alcohols (glycerol, ethanol) according to the procedure described by Calull *et al.* (1992). Amino acids were separated by ion-exchange chromatography on an anionic Ultropac-8 lithium-form resin (Amersham Pharmacia Biotech) with a Chromakon 400 (Kontron) and a Biochrom 20 analyzer (Amersham Pharmacia Biotech). Amino acids were detected by reaction with nihydrin. Ammonia was measured using the enzymatic method from Bergmeyer and Beutler (1990) based on its conversion into glutamate by the glutamate dehydrogenase.

Fermentation parameters of the parental strains were determined from five independent fermentations and from duplicated fermentations for the other strains (segregants, hemizygous strains). Metabolites were measured in five biological replicates for the parental strains and in duplicate for the segregants, except the amino acids, which were determined from a single fermentation.

### Gene expression analysis

Microarray hybridization and image analysis was performed at the Biochip Platform in Toulouse (http://biopuces.genotoul.fr). The microarrays were obtained by spotting in duplicate the 6308 oligonucleotides (70-mer) from the yeast genome Oligoset (Operon) onto UltraGAP chips. Gene expression was analyzed for five independent cultures of each parent and one culture of each segregant. RNA was isolated at the midtime of fermentation when half of the sugar present in the medium was consumed (equivalent to 45 g CO_2_), and then labeled with the Chipshot direct labeling and cleanup kit (Promega). Each sample labeled with the Cy3 dye was compared with a common reference pool labeled with Cy5 dye and composed of a mix of equivalent concentration of the RNA of each segregant of the population (star design). Hybridizations were done twice and thus technical replicates are available for each measurement. Data analysis was performed using the R 2.9.2 software and the limma package (R Development Core Team 2010; Smyth 2005, Smyth *et al.* 2005; Smyth and Speed 2003). Within-array normalization was performed using the print-tip-loess method followed by a quantile method for between-slide normalization. For each measurement, the two technical replicates were averaged. Gene expression between the two parental strains (2 × 5 microarrays) was compared using *t*-tests. Differentially expressed genes were defined by filtering on adjusted *P* = 0.01 to control the false discovery rate (FDR) (Benjamini *et al.* 2001). For linkage analysis in the population of segregants, transcripts with a low signal-to-noise ratio were filtered out: we required the mean red (Cy5) signal to be three times greater than the mean background signal (Cy5). The complete data set is available on the Gene Expression Omnibus database (accession number GSE26437) (Edgar *et al.* 2002). An analysis of the correlation between gene expression and phenotype parameters was carried out using the Spearman correlation coefficient and the associated statistical test, followed by a correction of the multiplicity using Benjamini and Hochberg procedure. We selected genes with correlation coefficients > 0.6 and adjusted *P* < 0.05 to control the FDR.

### Genotyping and linkage analysis of the population of segregants

Genomic DNA was isolated, fragmented, labeled, and hybridized to Affymetrix YGS98 microarrays as previously described (Winzeler *et al.* 1999). This procedure was applied three times for the parental strains S288c and 59A and one time for each of the 30 segregants. Probes with a unique match on the S288c genome were then screened to identify biallelic markers using the same statistical procedure as Brem *et al.* (2002). First, probes identified as low perfect-match (PM) *vs.* mismatch (MM) hybridizers to S288c genomic DNA were discarded. All further analyses were performed on normalized log(PM/MM) values. We selected probe pairs that had high hybridization differences between S288c and 59A using the Z and z statistics described in Brem *et al.* (2002). A second test based on a clustering algorithm was used to select probe pairs for evidence of 2:2 segregation in the segregating population. Genotypes were then inferred from cluster assignments as described in Brem *et al.* (2002). This resulted in the selection of 2465 probes. The probes were further filtered using the genomic sequences of the parental strains S288c and 59A. For each probe, the presence of polymorphisms in the 59A genome was confirmed. We used Illumina high-throughput sequencing to obtain SNP information for 59A. Genomic DNA of the strain 59A was sequenced using Illumina Genome Analyzer II technology (Illumina, Inc.) with paired reads of 36 bp using standard manufacturer protocols. Read sequences (deposited in the NCBI Sequence Read Archive as study SRP004704) were aligned to the S288c genome with MAQ (ver. 0.7.1) (Li *et al.* 2008) using default parameters to detect SNP. This filtering step resulted in a map of 1834 markers used in the linkage analysis.

For each phenotype and each expression trait, linkage analysis was performed using a normal model with the Haley-Knott regression method implemented in the R/qtl package (Broman *et al.* 2003). Logarithm of odds (LOD) scores were computed for each marker every 2.5 cM. An interval estimate of the location of each QTL or eQTL was obtained as the 1-LOD support interval: the region in which the LOD score is within 1 unit of the peak LOD score. Statistical significance was assessed by random permutation of the phenotypes or expression levels relative to the genotype data. For eQTL, the permutation was performed 10 times, and the average number of transcripts showing linkage at a specific LOD score was used to calculate FDR. For each phenotypic QTL, 1000 permutations were used to calculate an individual LOD score threshold and FDR.

### The GenYeasTrait resource

A database named GenYeasTrait, which is dedicated to the analysis of QTL and eQTL data, was developed using the Gmod/Gbrowse software version 2.15 (Stein *et al.* 2002). It includes the annotation of the S288c genome from the Saccharomyces Genome Database, SNPs detected from the comparison of the 59A strain sequence with the S288c reference genome, and the fermentation and expression QTL of the project. The database can be queried by using the Gbrowse interface, which allows user friendly graphical representations of features on chromosomes and facilitates data exploration. It is available at http://genome.jouy.inra.fr/genyeastrait/.

## Results

### Characterization of segregants from a cross between a derivative haploid of the wine yeast EC1118 and the laboratory strain S288C

To generate a population of segregants for QTL analysis, the S288C laboratory strain and the 59A strain (a haploid derivative of the EC1118 strain) were used for crossing (Figure 1). The fermentation profile of the haploid derivative 59A does not however perfectly overlap with the EC1118 pattern. Such variation is consistent with the known heterozygosity of the wine yeast EC1118 genome that was shown to contain 0.2% of heterozygous SNP (Novo *et al.* 2009). The hybrid (Z59S) obtained by crossing 59A with S288C displays a fermentation profile intermediate between the two parental strains (Figure 1). Fermentation rate profiles obtained in a synthetic medium (MS300) that mimics natural grape musts provided relevant criteria to characterize the fermentation capacity of the strains. As shown in Figure 1, the two parental strains exhibit very different fermentation profiles with a much higher maximum fermentation rate (R_max_) for 59A compared with S288c. Furthermore, the 59A strain also shows a higher fermentation rate at 50% of sugar utilization (R_50_) with a consequently shorter duration of fermentation duration (F_d_). All of these data are consistent with a higher fermentation capacity of the 59A wine strain derivative compared *vs.* the laboratory strain. Under such fermentation conditions, the growth phase is restricted to the period of increase of the fermentation rate until R_max_, and then cells are subsequently fermented in a stationary phase (Rossignol *et al.* 2003).

The zygote Z59S was used to generated a population of haploid segregants. The spores obtained had a low viability with 54% viable. The proportion of asci with four viable spores was low (only 15%), and a majority (around 50%) had three viable spores. We kept only one complete tetrad in the analyzed population, and the other segregants were selected from 20 different asci. The 30 haploid progenies where characterized under the fermentation conditions previously described. To describe the fermentation capacity, we considered the fermentation rate at three different stages of fermentation: R_max_, R_50_ (fermentation rate at 50% fermentation), and R_70_ (fermentation rate at 70% fermentation). Other fermentation traits, such as fermentation duration (F_d_) and cellular population (C_p_), were taken into account. We also considered metabolites, such as the amount of assimilated nitrogen (N_ass_), glycerol (gly), acetic acid (ace), succinic acid (suc) and pyruvic acid (pyr) (Table S1, Table S2, and Table S3). We observed a high variability in the segregants fermentation profiles (supporting information, Figure S1). Several traits values (R_max_, R_50_, gly, pyr, ace) were roughly normally distributed within the population (*P* > 0.05, Shapiro-Wilk normality test), supporting a polygenic determinism of these phenotypes (Figure S2). The amount of assimilated nitrogen and, to some extent, R_70_ and F_d_, displayed a bimodal distribution suggesting a possible control by a major locus. Furthermore, all of these parameters, except R_50_, displayed values outside the parental range. This transgressive segregation indicates that alleles with opposite effects were present in the parental strains.

**Figure 2:**
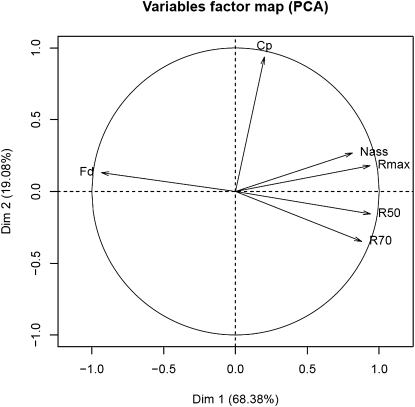
Principal component analysis (PCA) of kinetic and metabolic traits. This analysis shows that all kinetic parameters are correlated. The first component shows that N_ass_ and fermentation rates (R_max_, R_50_, R_70_) are negatively correlated with F_d_. The second component shows that C_p_ is not correlated with the other parameters.

To investigate potential relationships among fermentation traits, principal component analysis (PCA) was performed (Figure 2). The projection on two principal axes preserves 87% of the information and explains 68% and 19% of this variation, respectively. This analysis shows that F_d_, R_70_, and R_50_ are negatively correlated. This is consistent with the idea that the fermentation rate during the stationary phase strongly determines the overall duration of fermentation. The second axis also distinguishes the final cell population C_p_ and R_70_ variables (or C_p_ and R_max_) and the C_p_ and F_d_ variables which are not correlated. This suggests that the fermentation rate is independent from the total cell population. Finally, the fermentation rate at all stages (R_m_, R_50_, and R_70_) was positively correlated with the assimilated nitrogen N_ass_. These data indicate that cell capacity to use nitrogen has a strong impact on the fermentation rate. This is consistent with previous reports on the role of nitrogen availability in fermentation rate (Bely *et al.* 1990, Varela *et al.* 2004). The laboratory strain has a poor ability to consume nitrogen, which was associated to a weak fermentation capacity (Figure S1 and Figure S2).

### Transcripts abundance in parental strains and in the population of segregants

To assess the gene expression level of the entire genome of the segregants and parental strains, we performed a transcriptome analysis. The physiological condition chosen for analyzing gene expression was the point of midfermentation (50% of fermentation process). At this stage, cells are in stationary phase and are experiencing a general stress due to nutrient starvation and alcohol accumulation (about 6% at this stage). Under these physiological conditions, the transcriptome is stable and expected to provide a relevant picture regarding the capacity to resist to such stressful conditions (Rossignol *et al.* 2003). The transcriptomes of both parents and each segregant were compared with the mean transcriptome of the population (see *Materials and Methods*). We first observed that 2184 genes were differentially expressed between the parental strains at *P* < 0.01 and 262 genes up- or downregulated with a ratio higher than 2 (see *Materials and Methods*). This indicates that a large fraction of the genes in the genome are differentially expressed, with only a small subset displaying strong variations in agreement with previous reports (Brem *et al.* 2002). Among the top 100 genes that were strongly overexpressed in the parental strains (Table S3), 59A exhibited a large set of stress- and anaerobiosis-inducible cell wall genes from the *PAU* and *DAN* families. These genes are known to be highly expressed during alcoholic fermentation (Rossignol *et al.* 2003); however, given that several of them can cross-hybridize, it is not possible to identify unambiguously the set of genes really overexpressed. In the laboratory strain, there was a massive overexpression of 76 retrotransposons in agreement with a reshaping of retrotransposons in S288c and an amplification of Ty1 in the laboratory strain compared with EC1118 (Novo *et al.* 2009). Another striking feature in the laboratory strain was the strong overexpression of several genes involved in the stress response (*SPS100*, *HSP30*, *HSP12*).

### Correlations between gene expression and phenotypes

To address the relationships between fermentation phenotypes and gene expression, we carried out a correlation analysis (see *Materials and Methods*). The expression of a large number of genes exhibited significant correlation with kinetic parameters (Table 1 and Figure S4). Genes with the strongest correlation with R_max_ were involved in nitrogen metabolism (*MEP2*, *PUT1*, *MAE1*), protein synthesis and degradation (*MAP1*, *SUP35*, *PRE1*), or Ty protein. Several of these genes were also correlated with the amount of assimilated nitrogen N_ass_. Expression of these genes is controlled by nitrogen catabolic repression (NCR), and they are induced at the beginning of the stationary phase in response to nitrogen depletion (Rossignol *et al.* 2003). Their correlation with R_max_ and N_ass_ is consistent with their control by nitrogen availability and the role of nitrogen in fermentation rate (Figure 2 and Table 1). The genes that correlated with the fermentation rate at midfermentation (R_50_) and at 70% fermentation (R_70_) were very similar. Several genes involved in thiamine metabolism (*THI3*, *THI4*, and *PET18*) displayed a high level of correlation as did the actin gene *ACT1*, and a cell wall gene (*SCW4*). Correlation was also seen, though to a lesser extent, for genes involved in nitrogen metabolism (*MEP2*, *PUT1*, *MAP1*). The genes correlated with fermentation duration (F_d_) were mostly consistent with those that correlated with R_70_ or R_50_, but with the opposite relationship (these two traits are negatively correlated in PCA; see Figure 2). Peculiarly, this included various genes involved in the stress response (*CUP1*, *HSP12*, *HSP30*, *CTT1*, *SSA4*, *ATC1*) which were positively correlated with the fermentation duration F_d_, indicating that a high stress response was associated with a low fermentation capacity. Several members of the *PAU/DAN* family previously observed to be overexpressed in the industrial strain were correlated with nitrogen assimilation, and to some extent, with R_max_ and R_50_. For the other fermentation traits, such as cell population and amount of metabolites, weak or no correlation was found with gene expression. This was probably because the stage at which gene expression was analyzed (50% of fermentation progress) was not appropriate to address these parameters, which are mainly associated with the growth phase.

**Table 1:** Correlations between fermentation phenotypes and gene expression

Name	Correlation	P	Adjusted *P*	Function
Spearman correlations relative to R_max_				
MEP2	0.7456	2.27E−06	0.002	Ammonium permease involved in regulation of pseudohyphal growth
MAE1	0.6902	2.44E−05	0.01	Mitochondrial malic enzyme
PUT1	0.6833	3.16E−05	0.01	Proline oxidase
SNG1	0.6824	3.28E−05	0.01	Protein involved in nitrosoguanidine (MNNG) resistance
SUP35	0.6795	3.64E−05	0.01	Translation termination factor eRF3
YLR410W-B	0.6598	7.30E−05	0.013	Retrotransposon TYA Gag and TYB Pol genes
YGR038C-B	0.6452	1.18E−04	0.017	Retrotransposon TYA Gag and TYB Pol genes
YFL002W-A	0.6357	1.60E−04	0.018	Retrotransposon TYA Gag and TYB Pol genes
YBR016W	0.6272	2.08E−04	0.02	Plasma membrane protein of unknown function
YPR137C-B	0.6241	2.28E−04	0.02	Retrotransposon TYA Gag and TYB Pol genes
YCL076W	0.6239	2.30E−04	0.02	Dubious open reading frame unlikely to encode a protein
YLR035C-A	0.6208	2.52E−04	0.022	Retrotransposon TYA Gag and TYB Pol genes
RPN6	0.6117	3.29E−04	0.025	Essential
YML131W	0.6094	3.51E−04	0.026	Putative protein of unknown function with similarity to medium chain dehydrogenase/reductases
YDR261W-B	0.6038	4.11E−04	0.029	Retrotransposon TYA Gag and TYB Pol genes
YBL113C	−0.7505	1.79E−06	0.002	Helicase-like protein encoded within the telomeric Y element
YHR219W	−0.7487	1.95E−06	0.002	Putative protein of unknown function with similarity to helicases
YKL050C	−0.7429	2.58E−06	0.002	Protein of unknown function
YHR097C	−0.7414	2.78E−06	0.002	Putative protein of unknown function
YLR326W	−0.7409	2.84E−06	0.002	Putative protein of unknown function
YRF1-3	−0.7209	7.01E−06	0.004	Helicase encoded by the Y element of subtelomeric regions
YOR289W	−0.716	8.64E−06	0.005	Putative protein of unknown function
YJL225C	−0.6931	2.18E−05	0.01	Putative protein of unknown function
GFD1	−0.6902	2.44E−05	0.01	Coiled-coiled protein of unknown function
APM1	−0.6864	2.82E−05	0.01	Mu1-like medium subunit of the clathrin-associated protein complex (AP-1)
PHM7	−0.6848	2.99E−05	0.01	Protein of unknown function
YMR244C-A	−0.6775	3.91E−05	0.01	Putative protein of unknown function
MSC3	−0.6742	4.42E−05	0.011	Protein of unknown function
MSO1	−0.6713	4.90E−05	0.011	Probable component of the secretory vesicle docking complex
TMA23	−0.671	4.94E−05	0.011	Nucleolar protein of unknown function implicated in ribosome biogenesis
MRS1	−0.6693	5.26E−05	0.011	Protein required for the splicing of two mitochondrial group I introns (BI3 in COB and AI5beta in COX1)
CUP1-2	−0.6659	5.91E−05	0.012	Metallothionein
BNA1	−0.6591	7.47E−05	0.013	3-hydroxyanthranilic acid dioxygenase
IES5	−0.6589	7.53E−05	0.013	Protein that associates with the INO80 chromatin remodeling complex under low-salt conditions
VOA1	−0.6515	9.65E−05	0.015	Putative protein of unknown function
NSA1	−0.6512	9.72E−05	0.015	Constituent of 66S pre-ribosomal particles
YGR251W	−0.6486	1.06E−04	0.016	Essential protein required for maturation of 18S rRNA
CUP1-1	−0.6475	1.10E−04	0.016	Metallothionein
YPL080C	−0.6437	1.24E−04	0.017	Dubious open reading frame unlikely to encode a protein
PIN2	−0.6419	1.32E−04	0.017	Protein that induces appearance of [PIN+] prion when overproduced
BSC4	−0.6406	1.37E−04	0.017	Protein of unknown function
CSM2	−0.6397	1.41E−04	0.017	Protein required for accurate chromosome segregation during meiosis
YMR086W	−0.6392	1.43E−04	0.017	Protein of unknown function that may interact with ribosomes
YEL077C	−0.6386	1.46E−04	0.017	Helicase-like protein encoded within the telomeric Y element
COX16	−0.6357	1.60E−04	0.018	Mitochondrial inner membrane protein
YLL066C	−0.6352	1.62E−04	0.018	Putative protein of unknown function with similarity to helicases
OXR1	−0.6348	1.64E−04	0.018	Protein of unknown function required for normal levels of resistance to oxidative damage
YRF1-6	−0.6328	1.75E−04	0.018	Helicase encoded by the Y element of subtelomeric regions
YML133C	−0.629	1.97E−04	0.02	Putative protein of unknown function with similarity to helicases
DAL81	−0.6285	2.00E−04	0.02	Positive regulator of genes in multiple nitrogen degradation pathways
TOA1	−0.6268	2.11E−04	0.02	TFIIA large subunit
MDM12	−0.6254	2.19E−04	0.02	Mitochondrial outer membrane protein
CDC12	−0.6248	2.24E−04	0.02	Component of the septin ring of the mother-bud neck that is required for cytokinesis
YMR306C-A	−0.619	2.66E−04	0.022	Dubious open reading frame unlikely to encode a functional protein
VPS24	−0.6174	2.78E−04	0.023	One of four subunits of the endosomal sorting complex required for transport III (ESCRT-III)
DDC1	−0.6154	2.95E−04	0.024	DNA damage checkpoint protein
ATH1	−0.6139	3.09E−04	0.025	Acid trehalase required for utilization of extracellular trehalose
KES1	−0.6127	3.19E−04	0.025	Member of the oxysterol binding protein family
MEC3	−0.611	3.36E−04	0.025	DNA damage and meiotic pachytene checkpoint protein
PUP1	−0.6075	3.71E−04	0.027	Endopeptidase with trypsin-like activity that cleaves after basic residues
YDL173W	−0.6038	4.11E−04	0.029	Putative protein of unknown function
SPG1	−0.6028	4.23E−04	0.03	Protein required for survival at high temperature during stationary phase
Spearman correlations relative to R_50_				
THI3	0.7442	5.30E−06	0.003	Probable alpha-ketoisocaproate decarboxylase
THI4	0.7228	1.18E−05	0.006	Thiazole synthase
ACT1	0.7201	1.31E−05	0.006	Actin
SCW4	0.7152	1.57E−05	0.006	Cell wall protein with similarity to glucanases
YLR444C	0.7063	2.19E−05	0.007	Dubious open reading frame unlikely to encode a functional protein
HPA3	0.7032	2.45E−05	0.007	D-Amino acid N-acetyltransferase
SNG1	0.6866	2.79E−05	0.007	Protein involved in nitrosoguanidine (MNNG) resistance
TIM21	0.6845	4.75E−05	0.01	Constituent of the mitochondrial inner membrane presequence translocase (TIM23 complex)
PRE1	0.6783	5.87E−05	0.011	Beta 4 subunit of the 20S proteasome
PET18	0.6747	6.61E−05	0.012	Protein required for respiratory growth and stability of the mitochondrial genome
MAP1	0.6556	1.22E−04	0.018	Methionine aminopeptidase
MAE1	0.6534	1.31E−04	0.018	Mitochondrial malic enzyme
MEP2	0.6525	1.34E−04	0.018	Ammonium permease involved in regulation of pseudohyphal growth
YNR048W	0.6463	1.62E−04	0.021	Protein proposed to interact with phospholipid translocases
ERG20	0.6449	1.69E−04	0.021	Farnesyl pyrophosphate synthetase
LYS9	0.6387	2.03E−04	0.024	Saccharopine dehydrogenase (NADP+)
TPK1	0.6356	2.22E−04	0.025	cAMP-dependent protein kinase catalytic subunit
YAR069C	0.6343	2.31E−04	0.025	Dubious open reading frame unlikely to encode a protein
BUD31	0.6343	2.31E−04	0.025	Protein involved in bud-site selection
ARC1	0.6307	2.56E−04	0.025	Protein that binds tRNA and methionyl- and glutamyl-tRNA synthetases (Mes1p and Gus1p)
PAU1	0.6294	2.65E−04	0.025	Part of 23-member seripauperin multigene family encoded mainly in subtelomeric regions
YLR179C	0.6294	2.65E−04	0.025	Protein of unknown function
CDC45	0.6249	3.01E−04	0.027	DNA replication initiation factor
RPS21A	0.6236	3.12E−04	0.027	Protein component of the small (40S) ribosomal subunit
SOL2	0.6218	3.28E−04	0.028	Protein with a possible role in tRNA export
SSZ1	0.6151	3.93E−04	0.032	Hsp70 protein that interacts with Zuo1p (a DnaJ homolog) to form a ribosome-associated complex that binds the ribosome via the Zuo1p subunit
CDC7	0.6036	5.35E−04	0.038	DDK (Dbf4-dependent kinase) catalytic subunit required for firing origins and replication fork progression in mitosis through phosphorylation of Mcm2-7p complexes and Cdc45p
DRN1	0.6031	5.41E−04	0.038	Putative debranching enzyme associated ribonuclease
JSN1	0.6013	5.66E−04	0.04	Member of the Puf family of RNA-binding proteins
TVP23	−0.7913	1.42E−06	0.002	Integral membrane protein localized to late Golgi vesicles along with the v-SNARE Tlg2p
SPC1	−0.7878	1.51E−06	0.002	Subunit of the signal peptidase complex (SPC)
GCN3	−0.7709	2.21E−06	0.002	Alpha subunit of the translation initiation factor eIF2B
CUP1-2	−0.7677	2.42E−06	0.002	Metallothionein
YLL032C	−0.7602	3.04E−06	0.002	Protein of unknown function that may interact with ribosomes
HSP30	−0.7595	1.14E−06	0.002	Hydrophobic plasma membrane localized
CUP1-1	−0.7179	1.42E−05	0.006	Metallothionein
APM1	−0.7126	1.74E−05	0.006	Mu1-like medium subunit of the clathrin-associated protein complex (AP-1)
Q0182	−0.7006	2.70E−05	0.007	Dubious open reading frame unlikely to encode a protein
HSP12	−0.6988	2.88E−05	0.007	Plasma membrane localized protein that protects membranes from desiccation
ATG1	−0.693	3.54E−05	0.008	Protein ser/thr kinase required for vesicle formation in autophagy and the cytoplasm-to-vacuole targeting (Cvt) pathway
GND2	−0.693	3.54E−05	0.008	6-phosphogluconate dehydrogenase (decarboxylating)
ATC1	−0.6814	5.28E−05	0.011	Nuclear protein
YGR026W	−0.6814	5.28E−05	0.011	Putative protein of unknown function
YEL077C	−0.6756	6.41E−05	0.012	Helicase-like protein encoded within the telomeric Y element
CRS5	−0.6659	8.83E−05	0.014	Copper-binding metallothionein
RHO5	−0.6641	9.35E−05	0.015	Nonessential small GTPase of the Rho/Rac subfamily of Ras-like proteins
SPG1	−0.659	7.49E−05	0.013	Protein required for survival at high temperature during stationary phase
SPS100	−0.6512	1.40E−04	0.019	Protein required for spore wall maturation
YOR277C	−0.6412	1.34E−04	0.018	Dubious open reading frame unlikely to encode a protein
YOL014W	−0.6409	1.90E−04	0.023	Putative protein of unknown function
SSA4	−0.6343	2.31E−04	0.025	Heat shock protein that is highly induced upon stress
ADA2	−0.632	2.46E−04	0.025	Transcription coactivator
PIB2	−0.6298	2.62E−04	0.025	Protein binding phosphatidylinositol 3-phosphate
YNR047W	−0.6249	3.01E−04	0.027	Putative protein kinase that
INP53	−0.6249	3.01E−04	0.027	Polyphosphatidylinositol phosphatase
YPC1	−0.6218	3.28E−04	0.028	Alkaline ceramidase that also has reverse (CoA-independent) ceramide synthase activity
YJL114W	−0.6165	3.79E−04	0.031	Retrotransposon TYA Gag gene cotranscribed with TYB Pol
HXT8	−0.6102	4.49E−04	0.035	Protein of unknown function with similarity to hexose transporter family members
CYC1	−0.6085	4.70E−04	0.036	Cytochrome c
CTT1	−0.6067	4.93E−04	0.037	Cytosolic catalase T
ATP6	−0.605	3.98E−04	0.032	Mitochondrially encoded subunit a of the F0 sector of mitochondrial F1F0 ATP synthase
FCP1	−0.6049	5.16E−04	0.038	Carboxy-terminal domain (CTD) phosphatase
Spearman correlations relative to R_70_				
THI3	0.8427	5.18E−09	0	Probable alpha-ketoisocaproate decarboxylase
YLR444C	0.8028	9.39E−08	0	Dubious open reading frame unlikely to encode a functional protein
THI4	0.7817	3.40E−07	0	Thiazole synthase
RPS21A	0.7759	4.71E−07	0	Protein component of the small (40S) ribosomal subunit
PET18	0.7483	1.99E−06	0.001	Protein required for respiratory growth and stability of the mitochondrial genome
TPK1	0.7468	2.14E−06	0.001	cAMP-dependent protein kinase catalytic subunit
SCW4	0.7452	2.31E−06	0.001	Cell wall protein with similarity to glucanases
ACT1	0.741	2.83E−06	0.001	Actin
HPA3	0.7243	6.04E−06	0.001	D-Amino acid N-acetyltransferase
ERG20	0.7227	6.47E−06	0.001	Farnesyl pyrophosphate synthetase
TIM21	0.7165	8.45E−06	0.001	Constituent of the mitochondrial inner membrane presequence translocase (TIM23 complex)
DRN1	0.7112	1.06E−05	0.001	Putative debranching enzyme associated ribonuclease
PRE1	0.7109	1.07E−05	0.001	Beta 4 subunit of the 20S proteasome
YAR069C	0.6963	1.93E−05	0.002	Dubious open reading frame unlikely to encode a protein
ARR1	0.6951	2.02E−05	0.002	Transcriptional activator of the basic leucine zipper (bZIP) family
RPL14B	0.694	2.10E−05	0.002	Protein component of the large (60S) ribosomal subunit
CCT7	0.6931	2.18E−05	0.002	Subunit of the cytosolic chaperonin Cct ring complex
MAP1	0.6931	2.18E−05	0.002	Methionine aminopeptidase
RPL14A	0.6916	2.31E−05	0.002	N-terminally acetylated protein component of the large (60S) ribosomal subunit
CDC7	0.6871	2.74E−05	0.003	DDK (Dbf4-dependent kinase) catalytic subunit required for firing origins and replication fork progression in mitosis through phosphorylation of Mcm2-7p complexes and Cdc45p
BUD31	0.6862	2.83E−05	0.003	Protein involved in bud-site selection
LYS9	0.6851	2.95E−05	0.003	Saccharopine dehydrogenase (NADP+)
PDC5	0.6836	3.13E−05	0.003	Minor isoform of pyruvate decarboxylase
YLR179C	0.6836	3.13E−05	0.003	Protein of unknown function
SSZ1	0.6785	3.78E−05	0.003	Hsp70 protein that interacts with Zuo1p (a DnaJ homolog) to form a ribosome-associated complex that binds the ribosome via the Zuo1p subunit
CTS1	0.6707	5.00E−05	0.004	Endochitinase
YHB1	0.6575	7.87E−05	0.006	Nitric oxide oxidoreductase
HTA1	0.6569	8.05E−05	0.006	Histone H2A
POL30	0.6551	8.55E−05	0.006	Proliferating cell nuclear antigen (PCNA)
STR3	0.6547	8.68E−05	0.006	Cystathionine beta-lyase
VPS66	0.6493	1.04E−04	0.007	Cytoplasmic protein of unknown function involved in vacuolar protein sorting.
RPS10A	0.6455	1.17E−04	0.008	Protein component of the small (40S) ribosomal subunit
CYS4	0.6429	1.28E−04	0.008	Cystathionine beta-synthase
ARC19	0.6391	1.44E−04	0.009	Subunit of the ARP2/3 complex
HYP2	0.6375	1.51E−04	0.009	Translation initiation factor eIF-5A
RPL25	0.6373	1.52E−04	0.009	Primary rRNA-binding ribosomal protein component of the large (60S) ribosomal subunit
YNR048W	0.6326	1.76E−04	0.01	Protein proposed to interact with phospholipid translocases
CHO2	0.6315	1.82E−04	0.01	Phosphatidylethanolamine methyltransferase (PEMT)
THI21	0.6271	2.09E−04	0.011	Hydroxymethylpyrimidine phosphate kinase
RSC58	0.6242	2.27E−04	0.012	Component of the RSC chromatin remodeling complex
GFA1	0.6228	2.37E−04	0.012	Glutamine-fructose-6-phosphate amidotransferase
GEA1	0.619	2.65E−04	0.014	Guanine nucleotide exchange factor for ADP ribosylation factors (ARF)
SMF3	0.6177	2.76E−04	0.014	Putative divalent metal ion transporter involved in iron homeostasis
ZEO1	0.6153	2.96E−04	0.015	Peripheral membrane protein of the plasma membrane that interacts with Mid2p
DAL7	0.6128	3.18E−04	0.015	Malate synthase
ADH7	0.6117	3.29E−04	0.016	NADPH-dependent medium chain alcohol dehydrogenase with broad substrate specificity
LYS12	0.6095	3.50E−04	0.016	Homo-isocitrate dehydrogenase
JSN1	0.6075	3.71E−04	0.017	Member of the Puf family of RNA-binding proteins
YOX1	0.605	3.97E−04	0.018	Homeodomain-containing transcriptional repressor
CTA1	0.6046	4.02E−04	0.018	Catalase A
SPC1	−0.8714	3.72E−10	0	Subunit of the signal peptidase complex (SPC)
HSP30	−0.8505	2.66E−09	0	Hydrophobic plasma membrane localized
GCN3	−0.844	4.64E−09	0	Alpha subunit of the translation initiation factor eIF2B
TVP23	−0.824	2.21E−08	0	Integral membrane protein localized to late Golgi vesicles along with the v-SNARE Tlg2p
HSP12	−0.8238	2.24E−08	0	Plasma membrane localized protein that protects membranes from desiccation
ATG1	−0.8129	4.84E−08	0	Protein ser/thr kinase required for vesicle formation in autophagy and the cytoplasm-to-vacuole targeting (Cvt) pathway
GND2	−0.8033	9.13E−08	0	6-phosphogluconate dehydrogenase (decarboxylating)
SPS100	−0.7748	5.01E−07	0	Protein required for spore wall maturation
CRS5	−0.7639	9.03E−07	0	Copper-binding metallothionein
CUP1-2	−0.7632	9.35E−07	0	Metallothionein
Q0182	−0.7597	1.12E−06	0	Dubious open reading frame unlikely to encode a protein
CTT1	−0.7352	3.70E−06	0.001	Cytosolic catalase T
YLL032C	−0.7308	4.53E−06	0.001	Protein of unknown function that may interact with ribosomes
MSC1	−0.7295	4.80E−06	0.001	Protein of unknown function
SSA4	−0.7232	6.34E−06	0.001	Heat shock protein that is highly induced upon stress
INP53	−0.7212	6.92E−06	0.001	Polyphosphatidylinositol phosphatase
RHO5	−0.7172	8.22E−06	0.001	Nonessential small GTPase of the Rho/Rac subfamily of Ras-like proteins
ATP3	−0.7136	9.55E−06	0.001	Gamma subunit of the F1 sector of mitochondrial F1F0 ATP synthase
CUP1-1	−0.7127	9.91E−06	0.001	Metallothionein
ADA2	−0.7094	1.14E−05	0.002	Transcription coactivator
YPC1	−0.6983	1.78E−05	0.002	Alkaline ceramidase that also has reverse (CoA-independent) ceramide synthase activity
NCE102	−0.694	2.10E−05	0.002	Protein of unknown function
DDR2	−0.6931	2.18E−05	0.002	Multistress response protein
YJL114W	−0.6905	2.41E−05	0.002	Retrotransposon TYA Gag gene cotranscribed with TYB Pol
UFD1	−0.6891	2.54E−05	0.003	Protein that interacts with Cdc48p and Npl4p
ATP6	−0.6831	3.19E−05	0.003	Mitochondrially encoded subunit a of the F0 sector of mitochondrial F1F0 ATP synthase
APM1	−0.6814	3.40E−05	0.003	Mu1-like medium subunit of the clathrin-associated protein complex (AP-1)
HXT8	−0.672	4.77E−05	0.004	Protein of unknown function with similarity to hexose transporter family members
PMP3	−0.668	5.49E−05	0.004	Small plasma membrane protein related to a family of plant polypeptides that are overexpressed under high salt concentration or low temperature
FCP1	−0.6535	9.01E−05	0.007	Carboxy-terminal domain (CTD) phosphatase
YNR047W	−0.6529	9.21E−05	0.007	Putative protein kinase that
ATC1	−0.6509	9.84E−05	0.007	Nuclear protein
NDD1	−0.6504	9.98E−05	0.007	Transcriptional activator essential for nuclear division
SIP18	−0.6498	1.02E−04	0.007	Protein of unknown function whose expression is induced by osmotic stress
HXT13	−0.646	1.15E−04	0.008	Hexose transporter
CYC1	−0.6455	1.17E−04	0.008	Cytochrome c
YGR026W	−0.6449	1.20E−04	0.008	Putative protein of unknown function
SME1	−0.6386	1.46E−04	0.009	Core Sm protein Sm E
YLR132C	−0.6353	1.62E−04	0.01	Essential protein of unknown function
TOM7	−0.634	1.69E−04	0.01	Component of the TOM (translocase of outer membrane) complex responsible for recognition and initial import steps for all mitochondrially directed proteins
YNL190W	−0.63	1.91E−04	0.011	Cell wall protein of unknown function
TRP4	−0.6282	2.02E−04	0.011	Anthranilate phosphoribosyl transferase of the tryptophan biosynthetic pathway
SFT1	−0.6226	2.39E−04	0.012	Intra-Golgi v-SNARE
COB	−0.6199	2.58E−04	0.013	Cytochrome b, mitochondrially encoded subunit of the ubiquinol-cytochrome c reductase complex which includes Cobp
PGD1	−0.6155	2.94E−04	0.015	Subunit of the RNA polymerase II mediator complex
DCS2	−0.6142	3.06E−04	0.015	Nonessential
GIS3	−0.6121	3.24E−04	0.016	Protein of unknown function
YBR013C	−0.6086	3.59E−04	0.016	Putative protein of unknown function
NSR1	−0.6086	3.59E−04	0.016	Nucleolar protein that binds nuclear localization sequences
YOR277C	−0.6029	4.22E−04	0.019	Dubious open reading frame unlikely to encode a protein
FCY1	−0.6026	4.25E−04	0.019	Cytosine deaminase
JJJ2	−0.6012	4.42E−04	0.019	Protein of unknown function
Spearman correlations relative to F_d_				
CUP1-2	0.8062	7.56E−08	0.0003	Metallothionein
CUP1-1	0.7866	2.56E−07	0.0004	Metallothionein
YLL032C	0.7857	2.69E−07	0.0004	Protein of unknown function that may interact with ribosomes
APM1	0.777	4.43E−07	0.0005	Mu1-like medium subunit of the clathrin-associated protein complex (AP-1)
SPC1	0.7372	3.38E−06	0.0028	Subunit of the signal peptidase complex (SPC)
TVP23	0.7336	3.98E−06	0.0028	Integral membrane protein localized to late Golgi vesicles along with the v-SNARE Tlg2p
GCN3	0.7309	4.49E−06	0.0028	Alpha subunit of the translation initiation factor eIF2B
ATG1	0.7051	1.36E−05	0.006	Protein ser/thr kinase required for vesicle formation in autophagy and the cytoplasm-to-vacuole targeting (Cvt) pathway
HSP12	0.696	1.95E−05	0.006	Plasma membrane localized protein that protects membranes from desiccation
HSP30	0.695	2.02E−05	0.006	Hydrophobic plasma membrane localized
SPG1	0.6903	2.43E−05	0.0065	Protein required for survival at high temperature during stationary phase
ADA2	0.6884	2.61E−05	0.0065	Transcription coactivator
GND2	0.6797	3.61E−05	0.0076	6-phosphogluconate dehydrogenase (decarboxylating)
SSA4	0.6626	6.63E−05	0.0127	Heat shock protein that is highly induced upon stress
CYC1	0.6566	8.14E−05	0.0133	Cytochrome c
SPT7	0.6535	9.03E−05	0.0137	Subunit of the SAGA transcriptional regulatory complex
YHR219W	0.651	9.79E−05	0.0137	Putative protein of unknown function with similarity to helicases
YNL108C	0.6492	1.04E−04	0.0138	Putative protein of unknown function with similarity to Tfc7p and prokaryotic phosphotransfer enzymes
YPL108W	0.6466	1.13E−04	0.0147	Cytoplasmic protein of unknown function
YOR277C	0.6453	1.18E−04	0.0147	Dubious open reading frame unlikely to encode a protein
YGR026W	0.6434	1.25E−04	0.0147	Putative protein of unknown function
SIP18	0.6434	1.25E−04	0.0147	Protein of unknown function whose expression is induced by osmotic stress
Q0182	0.6417	1.33E−04	0.0148	Dubious open reading frame unlikely to encode a protein
OXR1	0.6358	1.59E−04	0.0171	Protein of unknown function required for normal levels of resistance to oxidative damage
SMD2	0.6343	1.67E−04	0.0175	Core Sm protein Sm D2
FCP1	0.6301	1.90E−04	0.0192	Carboxy-terminal domain (CTD) phosphatase
SMA1	0.6294	1.94E−04	0.0192	Protein of unknown function involved in the assembly of the prospore membrane during sporulation
YBL113C	0.6287	1.98E−04	0.0192	Helicase-like protein encoded within the telomeric Y element
YJL114W	0.6274	2.07E−04	0.0193	Retrotransposon TYA Gag gene cotranscribed with TYB Pol
INP53	0.621	2.51E−04	0.0221	Polyphosphatidylinositol phosphatase
CRS5	0.6198	2.59E−04	0.0221	Copper-binding metallothionein
SKS1	0.6196	2.61E−04	0.0221	Putative serine/threonine protein kinase
POP6	0.6161	2.90E−04	0.0221	Subunit of both RNase MRP
GIS3	0.6147	3.01E−04	0.0221	Protein of unknown function
FMP48	0.6143	3.05E−04	0.0221	Putative protein of unknown function
YHR097C	0.6143	3.05E−04	0.0221	Putative protein of unknown function
RHO5	0.6143	3.05E−04	0.0221	Nonessential small GTPase of the Rho/Rac subfamily of Ras-like proteins
TMA23	0.614	3.07E−04	0.0221	Nucleolar protein of unknown function implicated in ribosome biogenesis
YGL117W	0.6103	3.42E−04	0.0227	Putative protein of unknown function
SFT1	0.6103	3.42E−04	0.0227	Intra-Golgi v-SNARE
SPS100	0.6089	3.56E−04	0.0227	Protein required for spore wall maturation
MSC1	0.6079	3.66E−04	0.0227	Protein of unknown function
ALG11	0.602	4.32E−04	0.024	Alpha-1
SME1	0.602	4.32E−04	0.024	Core Sm protein Sm E
YDL144C	0.6018	4.35E−04	0.024	Putative protein of unknown function
DCS2	0.6016	4.37E−04	0.024	Nonessential
YOL014W	0.6009	4.46E−04	0.0241	Putative protein of unknown function
YJL156W-A	0.6005	4.51E−04	0.0241	Dubious open reading frame unlikely to encode a protein
THI3	−0.714	9.39E−06	0.0052	Probable alpha-ketoisocaproate decarboxylase
SCW4	−0.7056	1.33E−05	0.006	Cell wall protein with similarity to glucanases
TIM21	−0.6984	1.77E−05	0.006	Constituent of the mitochondrial inner membrane presequence translocase (TIM23 complex)
TPK1	−0.6953	2.00E−05	0.006	cAMP-dependent protein kinase catalytic subunit
SNG1	−0.6947	2.05E−05	0.006	Protein involved in nitrosoguanidine (MNNG) resistance
THI4	−0.6877	2.68E−05	0.0065	Thiazole synthase
YLR444C	−0.6862	2.84E−05	0.0066	Dubious open reading frame unlikely to encode a functional protein
PUT1	−0.6815	3.38E−05	0.0074	Proline oxidase
ACT1	−0.6739	4.45E−05	0.0089	Actin
MEP2	−0.661	7.00E−05	0.0128	Ammonium permease involved in regulation of pseudohyphal growth
HPA3	−0.6588	7.55E−05	0.0133	D-Amino acid N-acetyltransferase
RPS21A	−0.6577	7.84E−05	0.0133	Protein component of the small (40S) ribosomal subunit
DAN2	−0.6543	8.77E−05	0.0137	Cell wall mannoprotein with similarity to Tir1p
BUD31	−0.6515	9.65E−05	0.0137	Protein involved in bud-site selection
YLR179C	−0.6506	9.94E−05	0.0137	Protein of unknown function
PET18	−0.643	1.27E−04	0.0147	Protein required for respiratory growth and stability of the mitochondrial genome
ARC1	−0.6412	1.35E−04	0.0148	Protein that binds tRNA and methionyl- and glutamyl-tRNA synthetases (Mes1p and Gus1p)
PAU1	−0.6283	2.01E−04	0.0192	Part of 23-member seripauperin multigene family encoded mainly in subtelomeric regions
HRT3	−0.6221	2.43E−04	0.0221	Putative SCF-ubiquitin ligase F-box protein
CDC7	−0.6192	2.64E−04	0.0221	DDK (Dbf4-dependent kinase) catalytic subunit required for firing origins and replication fork progression in mitosis through phosphorylation of Mcm2-7p complexes and Cdc45p
RNR2	−0.6187	2.68E−04	0.0221	Ribonucleotide-diphosphate reductase (RNR)
YIL067C	−0.6183	2.71E−04	0.0221	Uncharacterized protein of unknown function
PRE1	−0.614	3.07E−04	0.0221	Beta 4 subunit of the 20S proteasome
URA7	−0.6118	3.27E−04	0.0227	Major CTP synthase isozyme (see also URA8)
DBP5	−0.6105	3.40E−04	0.0227	Cytoplasmic ATP-dependent RNA helicase of the DEAD-box family involved in mRNA export from the nucleus
PAU7	−0.6092	3.53E−04	0.0227	Part of 23-member seripauperin multigene family
ERG20	−0.6089	3.56E−04	0.0227	Farnesyl pyrophosphate synthetase
YAR069C	−0.6087	3.58E−04	0.0227	Dubious open reading frame unlikely to encode a protein
JSN1	−0.6083	3.63E−04	0.0227	Member of the Puf family of RNA-binding proteins
RPL23B	−0.6072	3.73E−04	0.0228	Protein component of the large (60S) ribosomal subunit
CHO2	−0.6045	4.03E−04	0.024	Phosphatidylethanolamine methyltransferase (PEMT)
CDC45	−0.6034	4.16E−04	0.024	DNA replication initiation factor
YNR048W	−0.6031	4.19E−04	0.024	Protein proposed to interact with phospholipid translocases
SOL2	−0.6016	4.37E−04	0.024	Protein with a possible role in tRNA export
GLN1	−0.6	4.57E−04	0.0241	Glutamine synthetase (GS)
Spearman correlations relative to N_ass_				
PAU7	0.6761	6.32E−05	0.019	Part of 23-member seripauperin multigene family
SNG1	0.6568	8.07E−05	0.019	Protein involved in nitrosoguanidine (MNNG) resistance
PUT1	0.6556	1.22E−04	0.023	Proline oxidase
DAN2	0.6547	1.25E−04	0.023	Cell wall mannoprotein with similarity to Tir1p
PAU8	0.6383	2.06E−04	0.027	Hypothetical protein
MEP2	0.6383	2.06E−04	0.027	Ammonium permease involved in regulation of pseudohyphal growth
PLB2	0.6245	3.05E−04	0.033	Phospholipase B (lysophospholipase) involved in phospholipid metabolism
LHP1	0.6214	3.32E−04	0.034	RNA binding protein required for maturation of tRNA and U6 snRNA precursors
PAU13	0.6182	3.62E−04	0.034	Putative protein of unknown function
PAU1	0.6182	3.62E−04	0.034	Part of 23-member seripauperin multigene family encoded mainly in subtelomeric regions
MOG1	0.6151	3.93E−04	0.034	Conserved nuclear protein that interacts with GTP-Gsp1p
PAU14	0.6142	4.03E−04	0.034	Hypothetical protein
PAU4	0.6133	4.13E−04	0.034	Part of 23-member seripauperin multigene family encoded mainly in subtelomeric regions
SRB7	0.6125	4.23E−04	0.034	Subunit of the RNA polymerase II mediator complex
PAU10	0.6071	4.87E−04	0.038	Hypothetical protein
CBR1	0.6027	5.47E−04	0.038	Microsomal cytochrome b reductase
YBR016W	0.6018	5.60E−04	0.038	Plasma membrane protein of unknown function
PEX29	0.6009	5.73E−04	0.039	Peroxisomal integral membrane peroxin
YKL050C	−0.8091	1.11E−06	0.005	Protein of unknown function
MRS1	−0.7499	4.30E−06	0.009	Protein required for the splicing of two mitochondrial group I introns (BI3 in COB and AI5beta in COX1)
YBL113C	−0.7344	7.64E−06	0.01	Helicase-like protein encoded within the telomeric Y element
YHR097C	−0.7286	9.51E−06	0.01	Putative protein of unknown function
YJL225C	−0.7152	1.57E−05	0.014	Putative protein of unknown function
YMR086W	−0.709	1.98E−05	0.015	Protein of unknown function that may interact with ribosomes
YGR251W	−0.6974	3.02E−05	0.018	Essential protein required for maturation of 18S rRNA
COX16	−0.6948	3.32E−05	0.018	Mitochondrial inner membrane protein
TOA1	−0.6881	4.20E−05	0.019	TFIIA large subunit
YPL080C	−0.6823	5.12E−05	0.019	Dubious open reading frame unlikely to encode a protein
PEX14	−0.6796	5.61E−05	0.019	Peroxisomal membrane peroxin that is a central component of the peroxisomal protein import machinery
DDC1	−0.6739	6.80E−05	0.019	DNA damage checkpoint protein
YRF1-3	−0.6734	6.90E−05	0.019	Helicase encoded by the Y element of subtelomeric regions
YLL066C	−0.6707	7.54E−05	0.019	Putative protein of unknown function with similarity to helicases
YLR326W	−0.669	7.99E−05	0.019	Putative protein of unknown function
TMA23	−0.6672	8.46E−05	0.019	Nucleolar protein of unknown function implicated in ribosome biogenesis
PHM7	−0.6659	8.83E−05	0.019	Protein of unknown function
IES5	−0.6637	6.38E−05	0.019	Protein that associates with the INO80 chromatin remodeling complex under low-salt conditions
NSA1	−0.6636	9.48E−05	0.019	Constituent of 66S pre-ribosomal particles
MTF1	−0.6515	9.65E−05	0.019	Mitochondrial RNA polymerase
YMR244C-A	−0.6507	1.42E−04	0.023	Putative protein of unknown function
MDM12	−0.6503	1.44E−04	0.023	Mitochondrial outer membrane protein
YMR306C-A	−0.6489	1.50E−04	0.023	Dubious open reading frame unlikely to encode a functional protein
ERG24	−0.6489	1.50E−04	0.023	C-14 sterol reductase
ENP2	−0.6485	1.52E−04	0.023	Essential nucleolar protein of unknown function
AIM23	−0.6458	1.64E−04	0.024	Putative protein of unknown function
YHR219W	−0.6392	2.00E−04	0.027	Putative protein of unknown function with similarity to helicases
YDL173W	−0.6374	2.11E−04	0.027	Putative protein of unknown function
VOA1	−0.6352	2.25E−04	0.027	Putative protein of unknown function
CUE5	−0.6352	2.25E−04	0.027	Protein containing a CUE domain that binds ubiquitin
YOR021C	−0.6316	2.49E−04	0.029	Putative protein of unknown function
YAR028W	−0.6298	2.62E−04	0.03	Putative integral membrane protein
GFD1	−0.6267	2.86E−04	0.031	Coiled-coiled protein of unknown function
JID1	−0.6263	2.14E−04	0.027	Probable Hsp40p cochaperone
SCM3	−0.6209	3.36E−04	0.034	Nonhistone component of centromeric chromatin that binds stoichiometrically to CenH3-H4 histones
YLR363W-A	−0.6205	3.40E−04	0.034	Putative protein of unknown function
YOR289W	−0.616	3.84E−04	0.034	Putative protein of unknown function
IRC25	−0.6147	3.98E−04	0.034	Component of a heterodimeric Poc4p-Irc25p chaperone involved in assembly of alpha subunits into the 20S proteasome
TPK3	−0.6142	4.03E−04	0.034	cAMP-dependent protein kinase catalytic subunit
HCA4	−0.6133	4.13E−04	0.034	Putative nucleolar DEAD box RNA helicase
SGF11	−0.6102	4.49E−04	0.036	Integral subunit of SAGA histone acetyltransferase complex
YRF1-8	−0.608	4.76E−04	0.037	One of several telomeric Y element-encoded DNA helicases
CUS1	−0.6044	5.22E−04	0.038	Protein required for assembly of U2 snRNP into the spliceosome
GLC8	−0.6031	5.41E−04	0.038	Regulatory subunit of protein phosphatase 1 (Glc7p)
YML133C	−0.6027	5.47E−04	0.038	Putative protein of unknown function with similarity to helicases
SPO13	−0.6018	5.60E−04	0.038	Meiosis-specific protein
ELA1	−0.6004	5.79E−04	0.039	Elongin A

For each gene, the *P* value, the adjusted *P* value (Benjamini and Hochberg adjustment), and function are indicated.

### Marker map construction and identification of QTL for fermentation parameters

To genotype the entire population of this study, we hybridized the genomic DNA of the 30 segregants and parental strains on high-density olignonucleotide microarrays Affymetrix YGS98 (Winzeler *et al.* 1998). Using the genome sequences of the two strains, we obtained a dense coverage of the genome with 1834 physical markers and an average spacing of 6.6 kbp between each marker (Figure S5). The markers are expected to be reliable as we checked that each marker corresponded to a SNP in the genome sequence of the strain 59A. The coverage of the genome was rather homogeneous, with only few gaps on chromosome III and on the right arm of chromosome XIV. These regions of low coverage also have low SNP density that may explain the reduced frequency of markers (see Gbrowse at http://genome.jouy.inra.fr/genyeastrait/). The distribution of the markers in the 30 segregants was used to build a recombination map (Figure S6). Only very few chromosomes did not display crossover. We used the single tetrad to estimate the number of crossovers and found a frequency of 70 crossovers per meiosis, a number close to previous reports (Brem *et al.* 2002; Cubillos *et al.* 2011). The parental genomes markers were evenly distributed in the population, with 49.8% originating from the strain 59A and 50.2% from S288C.

**Figure 3:**
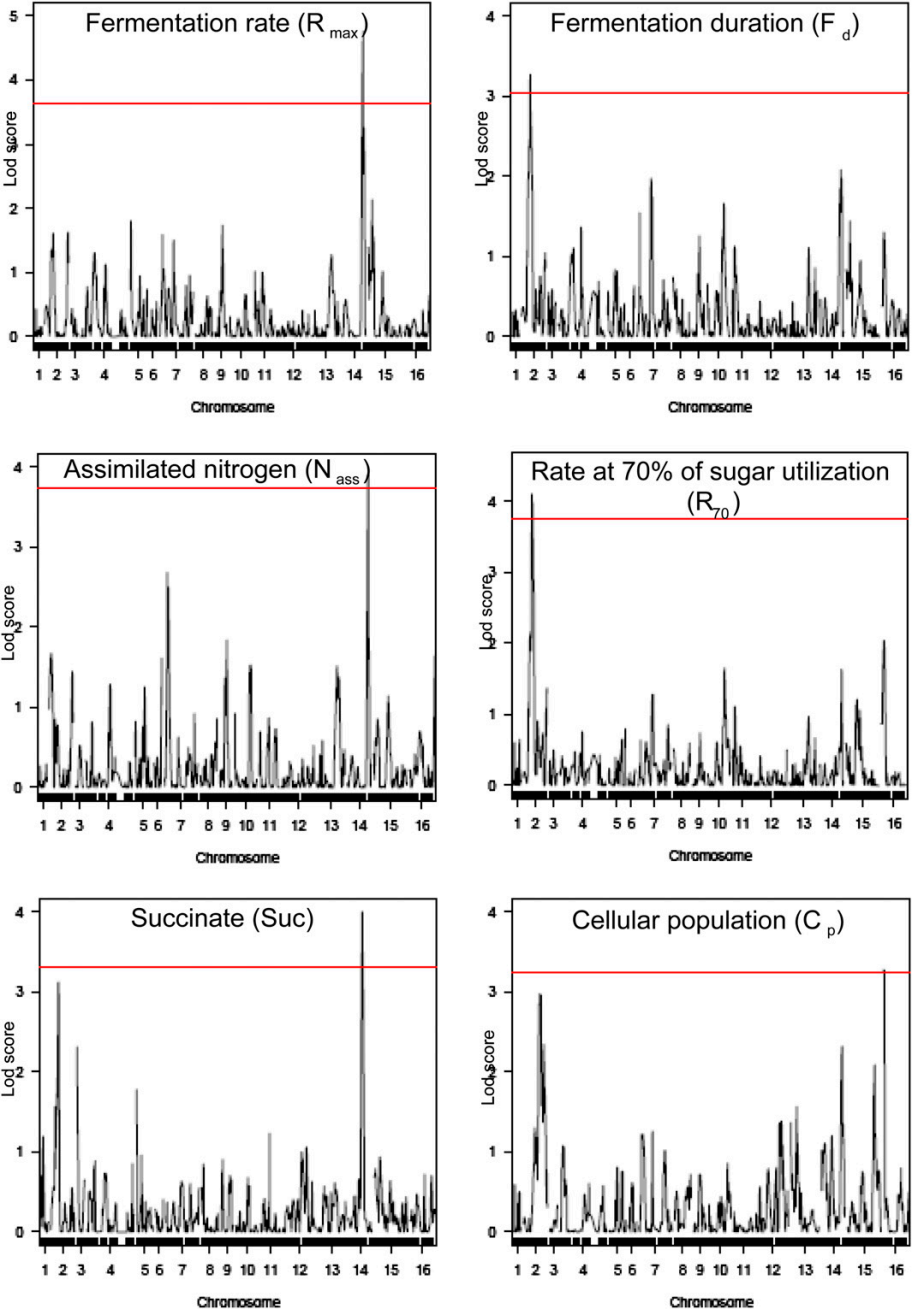
QTL mapping of kinetics traits and succinate production. The concatenated chromosomes are displayed on the X-axis and LOD score values on the Y-axis. The significant LOD score thresholds are indicated by a red line. Each peak of the LOD curve corresponds to the LOD value of linkage between a marker located in the X position and the value of each trait. Further details of QTL are in Table 2.

We then used these markers to map QTL for the different fermentation and metabolic parameters using an interval mapping approach (see *Materials and Methods*). We obtained a significant LOD score threshold for six different phenotypes. QTL were detected for R_max_ on chromosome XIV, for the cell population C_p_ on chromosome XVI, for the assimilated nitrogen N_ass_ on chromosome XIV, and for the fermentation duration F_d_ and R_70_ on chromosome II (Figure 3). A QTL for metabolite levels was found only for succinate production with a clear QTL located on chromosome XIV and one additional potential QTL, with a LOD score below the threshold, on chromosome II (Figure 3). We observed that QTL for various fermentation traits overlapped (Table 2). This concerned a QTL for the maximum fermentation rate and a QTL for the assimilated nitrogen that overlapped in a 37.5 kb region on chromosome XIV (664875 kb to 702375 kb). These results are consistent with the previous PCA results that highlighted relationships between nitrogen utilization N_ass_ and R_max_ (Figure 2). Two other parameters, R_70_ and F_d_, mapped to a common region on chromosome II, whereas R_50_ and succinate production had a potential linkage on the same region with a LOD score just below the threshold. Careful examination of the LOD score profiles suggests that the QTL for succinate is distinct from the R_70_ QTL (Figure S7). The overlap of QTL for R_70_, R_50_, and F_d_ is consistent with the known correlations between these parameters.

**Table 2:** Chromosomal location and size of the different phenotypic QTL

Traits	Chromosome	Size (kb)	Start Position (pb)	End Position (pb)	LOD Threshold	LOD Score
R_max_	XIV	37.50	664875	702375	3.64	4.76
N_ass_	XIV	37.50	664875	702375	3.73	3.91
F_d_	II	69.74	246162	315903	3.05	3.27
R_50_	II	69.74	246162	315903	3.74	3.11
Succinate	II	43.58	227328	270903	3.31	3.12
R_70_	II	60.00	263403	323403	3.75	4.1
C_p_	XVI	37.16	25617	62772	3.24	3.27
Succinate	XIV	60.00	447375	507375	3.31	4.01

The gray colors in the table indicate QTL that are overlapping. The first group of overlapping QTL, which is located on chromosome XIV, involved the maximum fermentation rate variation (R_max_) and nitrogen assimilation (N_ass_). The other group, which is localized on chromosome II, involves four traits as the succinate production, the fermentation duration, and the fermentation rate at 50% and 70% of sugar consumption. Two QTL mentioned (R_50_ and succinate on chromosome II) have a maximum LOD score just below the threshold but are conserved because they overlap with F_d_ and R_70_ QTL on chromosome II.

### Identification of expression QTL

In parallel, to identify loci controlling gene expression, the yeast genome was scanned for markers by gene expression linkages. We treated the expression signals from the whole data set of 4398 genes as quantitative traits without additional filtering and subjected them to linkage analysis using the set of markers. In doing so, a test statistic evaluating each marker-by-gene association was computed to detect the number of eQTL with different LOD score thresholds. For each significance threshold we then estimated a corresponding FDR using a permutation approach (see *Materials and Methods*). Depending on the LOD score threshold used, we could detect from 92 to 897 eQTL. Ninety-two eQTL were detected with a LOD score threshold of 4.5 (FDR of 0.26). A less stringent LOD score threshold of 3.5 (FDR of 0.50) was associated with 409 eQTL, and a LOD score threshold of 3.0 (FDR of 0.63) was associated with 897 eQTL. The high FDR associated with LOD scores was not surprising given the small population size of segregants used in our analysis. Indeed, all previous eQTL studies in yeast were performed using larger population sizes (Brem and Kruglyak 2005).

**Figure 4:**
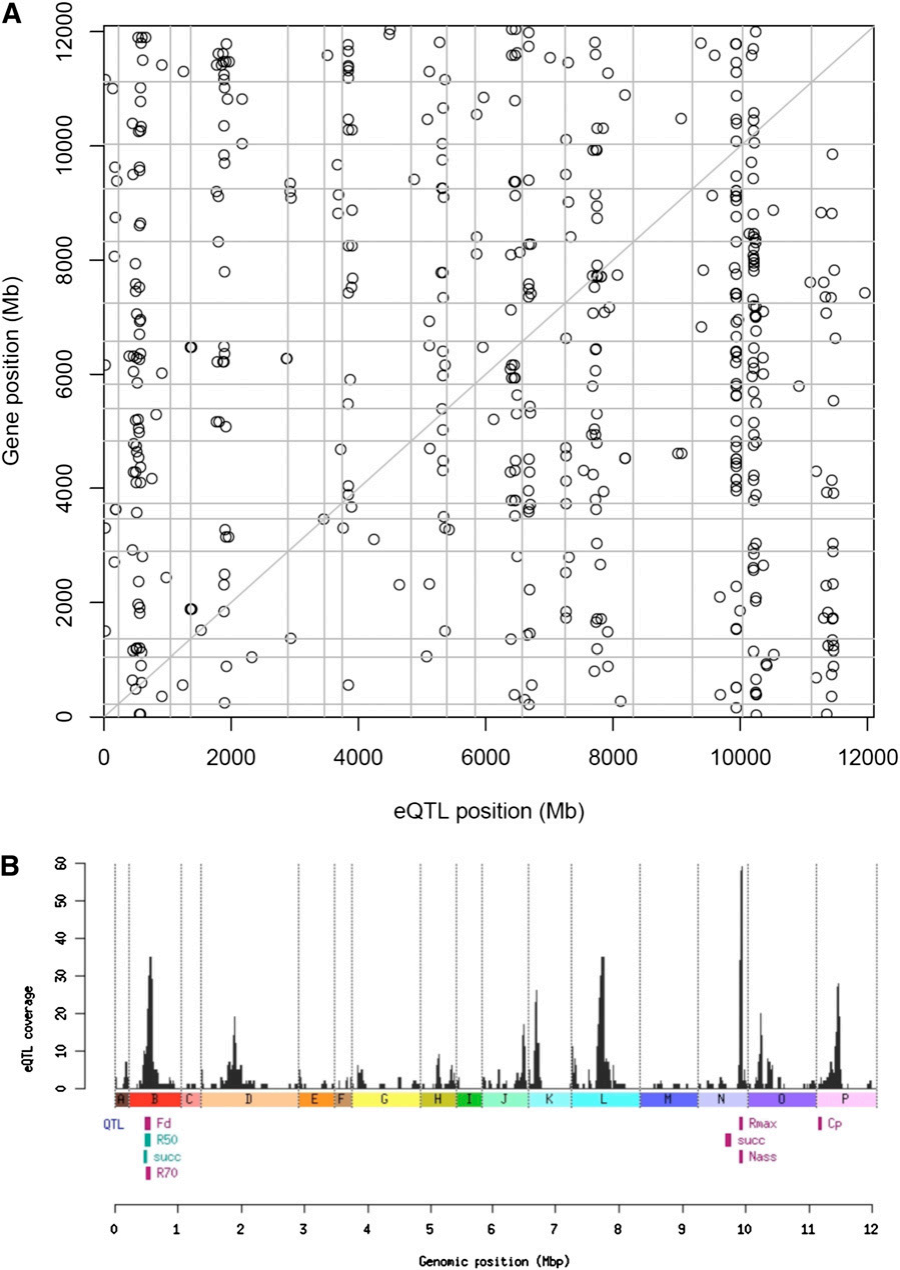
Genomic view of eQTL distribution and relationships with QTL for fermentation traits. A) Genomic view of eQTL mapping. The X-axis represents the genome location of markers, and the Y-axis represents the genome location of the regulated linked genes on concatenated chromosomes. EQTL values with LOD scores greater than 3.5 are displayed. The diagonal pattern is called “*cis*-eQTL band” and represents an association between the expression level of a gene and the genotype at the gene’s locus. Multiple vertical bands, called “*trans*-eQTL bands,” illustrate associations between the expression of many genes and a single locus. B) Overlapping of eQTL and QTL for fermentation traits. The scale below the figure indicates the genomic position across the genome in mega base pairs (Mpb). We can observe the overlapping of the hotspot on chromosome II with F_d_, R_70_, R_50_, and succinate (succ) parameters and the overlap of the hotspot located on chromosome XIV with R_max_ and N_ass_. QTL for traits with LOD scores just below the threshold are shown in blue.

The main interest in this study was to use the QTL analysis to gain an initial insight into the eQTL architecture and its relationship with fermentation QTL. We therefore decided to analyze the data set using a permissive LOD score threshold of 3.5. The physical location of eQTL across the genome was plotted against the physical location of genes that were differentially deregulated and possessed an eQTL using this LOD score (Figure 4). The point in the diagonal indicates *cis*-linkages, *i.e.*, meaning that the QTL linkage peak coincides with the physical location of the open reading frame for the expression trait. All of the eQTL located outside this diagonal represent *trans*-linkages with a linkage peaks at loci distant from the physical location of the open reading frames. We detected only a limited number, eight, of *cis*-associations visible on the diagonal line in Figure 4A. Among them, four ASP3 genes (*ASP3-1, ASP3-2, ASP3-3,* and *ASP3-4*) displayed a local regulation consistent with the known deletion of these genes in the industrial strain (Novo *et al.* 2009). Surprisingly, by observing within *trans*-eQTL distribution, we noticed the linkage of many transcripts to the same chromosomal region, which gave rise to vertical alignments of eQTL (Figure 4A). Seven such eQTL *trans*-bands could be observed and localized on chromosomes II, IV, XI, XII, XIV, XV and XVI. This distribution of eQTL corresponds to hotspot regions, which contain loci exerting a *trans*-control on the expression of a large set of genes (Figure 4A). These trans-regulatory bands were also visible at more stringent threshold levels (Figure S8), and this strengthens the notion of a role of these loci in the variation of expression of a large set of transcripts. The number of eQTL linked to these regions ranged from 12 on chromosome XVI to 42 on chromosome XIV (Figure 4 and Table S4). The position of individual eQTL can be examined using the Gbrowse interface at http://genome.jouy.inra.fr/genyeastrait/.

### Overlap between eQTL and QTL for fermentation traits

To determine whether the chromosomal loci detected in the QTL/eQTL analysis have an impact both on fermentation phenotypes and the massive deregulation of gene expression we examined the possible relationships between eQTL and QTL of fermentation phenotypes. Figure 4B displays the positions of fermentation QTL and the number of eQTL detected across the genome to visualize the hotspots in parallel. Among the seven detected eQTL hotspot regions, two overlap with fermentation QTL. The hotspot located on chromosome II overlaps with QTL involved in the variations of F_d_, R_70_, and succinate production. The 63 kb hotspot on chromosome XIV overlaps with QTL associated with variations in R_max_ and N_ass_ (Figure 4B). The colocalization of the two fermentation QTL with the eQTL hotspots suggests that a common regulator affects both the expression of the set of genes and the fermentation traits. As variations in parameters such as nitrogen assimilation are expected to be associated with strong physiological changes, their coupling with massive changes in gene expression is unsurprising. In addition, some genes, whose expression was previously found to be correlated with the fermentation parameters, are linked to the same hotspot (*e.g.*, the stress-responsive genes *HSP12*, *HSP30*, and *SPS100* are correlated with F_d_ and R_70_ and have a linkage on the chromosome-II hotspot).

### Dissection of the eQTL hotspot associated to the variation of R_m_ and N_ass_

**Figure 5:**
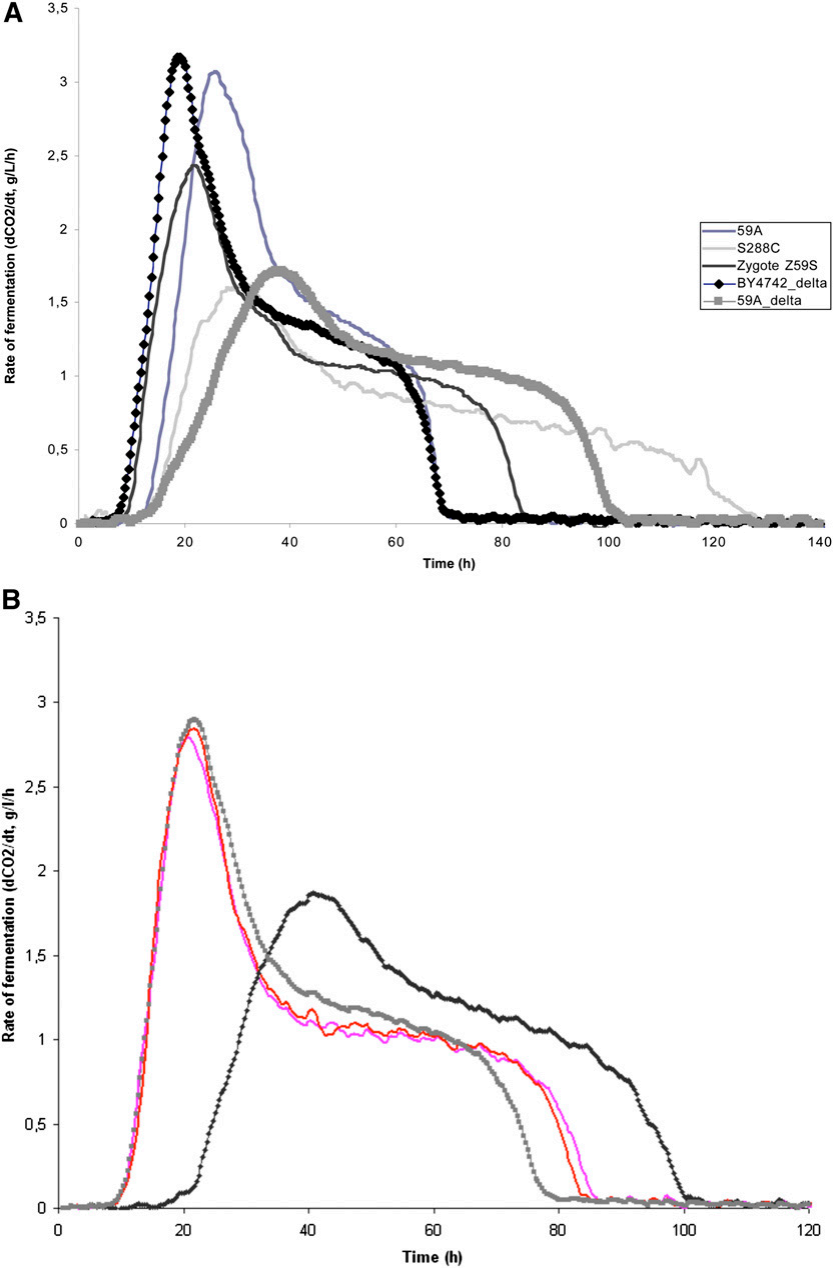
Reciprocal hemizygous analysis of ABZ1 and impact of *p*-aminobenzoate on the fermentation profiles. A) Fermentation rate profiles of the two reciprocal hemizygous strains carrying the *ABZ1* allele from BY4742 or 59A. The hemizygous strains harbor either an active *ABZ1* BY allele (strain BY4742/*ABZ1*-59A∆, thick gray line) or the 59A allele (strain 59A/*ABZ1*- BY4742∆, black line with diamonds). Profiles of the strains 59A (dark-blue line), the laboratory strain S288C (thin gray line), and the hybrid Z59S (black line) are shown. B) Impact of *p*-aminobenzoate on the fermentation profiles of hemizygous strains. Fermentation kinetics of two hemizygous strains in MS300 supplemented or not with 1 mg/l of *p*-aminobenzoic acid. Hemizygous strain carrying S288c *ABZ1* allele in MS300 without (black line) or supplemented with *p*-aminobenzoic acid (gray kinetic line). Hemizygous strain carrying 59A *ABZ1* allele in MS300, without (red kinetic line) or supplemented with *p*-aminobenzoic acid (pink kinetic line). The supplementation with *p*-aminobenzoic acid improves the fermentation capacity of the hemizygous strain carrying the S288c *ABZ1* allele.

We explored the hotspot covering the region from 635 kbp to 732 kbp on chromosome XIV to identify putative candidate genes that could be involved in the control of R_max_ and N_ass_. We used the genome sequence of the 59A strain to search for genes with either nonsynonymous SNP leading to amino acid changes in the coding region or with SNPs in the regulatory region and a self-eQTL linkage in the hotspot. Genes potentially connected to nitrogen metabolism were preferentially considered. We found a relevant candidate gene, *ABZ1*, which codes for a *p*-aminobenzoate synthase. This gene harbors 13 SNPs in the coding sequence and 5 of these are nonsynonymous substitutions (see http://genome.jouy.inra.fr/genyeastrait). *ABZ1* is also involved in methyl donor synthesis required for biosynthesis of methionine and nucleotides. The *ABZ1* gene is therefore connected to the nitrogen biosynthetic pathways and was considered to be worth for further investigations. We used a reciprocal-hemizygosity analysis described by Steinmetz *et al.* (2002) to investigate the impact of the *ABZ1* allele on the fermentation phenotype. We constructed an isogenic pair of strains by crossing 59A with BY4742Δabz1 on one side and a 59A strain, in which the *ABZ1* gene was deleted (59AΔabz1), with BY4742 on the other side. The two diploid strains differ only in their *ABZ1* alleles. In each strain, only one allele of *ABZ1* is functional, originating from either BY4742 or 59A. The fermentation profiles obtained with these two strains revealed that the *ABZ1* allele has a strong impact on fermentation behavior (Figure 5A). R_max_ was strongly decreased whereas F_d_ was increased when the hemizygous strain carried the *ABZ1* allele originating from BY4742. These results are a first confirmation of the implication of *ABZ1* in the control of the fermentation rate. The lower fermentation rate with the *ABZ1* allele from the BY4742 strain suggests that this allele could be associated with a defect in *p*-aminobenzoate synthesis. Actually, the synthetic medium used (MS300) (see *Materials and Methods*) did not contain *p*-aminobenzoate. We therefore examined how the fermentation kinetics of the hemizygous strains were altered following supplementing the medium with *p*-aminobenzoate. Indeed, when *p*-aminobenzoate was added (1 mg/l) to the fermentation medium, the hemizygous strain carrying the BY4742 *ABZ1* allele recovered a high fermentation rate (Figure 5B). In contrast, supplementation with *p*-aminobenzoate had no effect on the reciprocal strain carrying the 59A allele. These data are consistent with the fermentation rate being modulated by a limitation in *p*-aminobenzoate biosynthesis due to the presence of *ABZ1* allele of the laboratory strain. Since the QTL for N_ass_, overlapped with the QTL for R_max_, we checked whether N_ass_ was modulated by the *ABZ1* allele. An amino-acid analysis of the fermentation medium revealed that residual assimilable nitrogen was 30 mg/l with the strain carrying the *ABZ1* allele from BY4742 *vs.* 11 mg/l with the hemizygous strain containing the 59A form. These results indicate that nitrogen assimilation is also modulated by the *ABZ1* allele.

## Discussion

**Figure 6:**
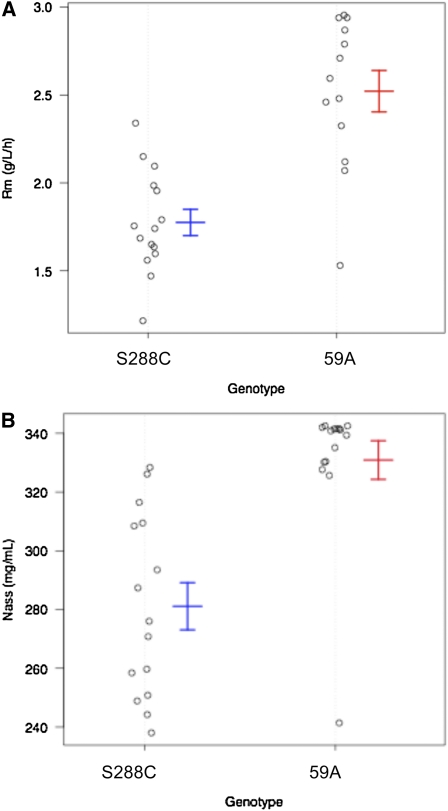
Impact of the *ABZ1* genotype on the fermentation phenotypes R_max_ and N_ass_ of the 30 segregants. A) Fermentation rate R_max_ of the segregants that inherited the *ABZ1* locus from S288C or 59A. B) Nitrogen assimilation (N_ass_) phenotype of the segregants that inherited the *ABZ1* locus from S288C or 59A. The average phenotype and standard deviation are indicated for each genotype group.

The genetic determinants of variation for most of the fermentation parameters in yeast are still unknown. In this study, we addressed the genetic basis of several fermentation traits and combined this with gene-expression analysis and an eQTL approach. Using a segregating population of a limited size we identified QTL for several fermentation traits. Accurate measures of kinetic parameters—specifically fermentation rates—allowed us to show that depending on the progress of fermentation, different QTL are associated with different fermentation rates; one for R_max_, and one for R_70_. This is consistent with the notion that, depending on fermentation progress, growth, or starvation, different cellular mechanisms are critical in controlling fermentation flux. On the other hand, these parameters share other components of cell metabolism, which is indicated by their high correlation and strong association with nitrogen utilization. Indeed, we found that R_max_ and N_ass_ shared a common QTL. This QTL was dissected, and we provided functional evidence that a gene involved in *p*-aminobenzoate synthesis plays a role in controlling R_max_ and N_ass_. The *ABZ1* allele has a strong effect on the fermentation rate and on nitrogen utilization as visualized in Figure 6. We calculated that *ABZ1* could explain 51% of the variance of R_max_ and 45% of the variance of N_ass_ in the segregants population.

The association of a transcriptomic analysis of the segregants population to a classical QTL approach has provided an important value of the study. Given the small size of the population, these data did not intend to decipher the regulatory variations globally. However, they could help at elucidating fermentation QTL and offer new insights into their relationships with gene expression. An outstanding observation was the evidence of overlaps among several fermentation QTL and eQTL hotspots. Two out of the seven identified hotspots overlapped with phenotypes. The other hotspots were not associated with phenotypic QTL. However, only a very small number of phenotypes were investigated, and examination of additional phenotypes is required to address potential associations with other hotspots. Similar associations between eQTL hotspots and phenotypes have been reported in yeast (Yvert *et al.* 2003) and other organisms (Fu *et al.* 2009). They are thought to originate from sequence variations that have broad pleiotropic effects. In the model plant, *Arabidopsis thaliana*, variation of complex quantitative traits could be explained by a few hotspot genomic regions that controlled various parameters (Fu *et al.* 2009). In the present study, we show that the *ABZ1* allele has a strong impact on the fermentation capacity of the strain. Indeed, such physiological changes are expected to be coupled to massive changes in gene expressions, which can explain the hotspot. In this case, the hotspot results from the effects of a strong metabolic alteration and not directly from modification of a general regulator.

Our data show that the *ABZ1* allele from S288c is unable to support an R_max_ that is similar to the industrial form. Because this phenomenon is abolished by the addition of *p*-aminobenzoate to the fermentation medium, we can infer that a limitation in the flux of this metabolite is responsible for the lower fermentation rate. *p*-aminobenzoate is an intermediate of the tetrahydrofolate biosynthetic pathway, which leads to a family of cofactors required for one-carbon transfer reactions. These methyl-donor compounds are involved in the synthesis of methionine, serine, and purines (Thomas and Surdin-Kerjan 1997). A limiting flux in *p*-aminobenzoate can therefore trigger a limitation in the availability of one of these metabolites. Given the pivotal role of methionine in translation, this may limit the rate of protein synthesis and in turn the incorporation of nitrogen sources. This mechanism is consistent with the observed correlation between R_max_ and N_ass_, as well as with the overlap of their QTL. Our results highlight the critical role of methyl-donor biosynthesis in the control of nitrogen utilization during alcoholic fermentation. Interestingly, we did not observe any significant effect of the *ABZ1* allele on the growth rate of the hemizygous strains (data not shown). This suggests that the methyl-donor pathway is more critical for sugar flux then for cell growth. The difference in nitrogen utilization triggered by the *ABZ1* alleles is also clearly responsible for the differential expression of genes regulated by nitrogen sources. This explains the correlations of the genes under NCR control, such as *MEP2* and *PUT1*, with R_max_. Most of the genes linked to the hotspot are however not involved in nitrogen metabolism but respond to various environmental changes including nitrogen depletion (Gasch *et al.* 2000). This suggests that they correspond to both direct and indirect effects of the *ABZ1* allele. Unexpectedly, we did not observe any deregulation of the *ABZ1* gene itself; it was not differentially expressed in the parental strains and displayed no change in the segregants. A similar lack of differential expression was also noticed for genes downstream *ABZ1* in the biosynthetic pathway, *ABZ2* and *FOL1* (data not shown). This suggests that the genes of the pathway are not or poorly regulated by the Abz1 product, 4-amino-4-deoxychorismate, or by downstream metabolites such as *p*-aminobenzoate.

The Abz1 protein from the strain 59A contains five amino acids changes compared with the S288c form. Comparison with the Abz1sequence of *Saccharomyces cerevisiae* strains from various origins available at the Sanger Institute (Liti *et al.* 2009) or in Genbank shows that the same changes are found in other wine yeasts, such as VIN13 (Borneman *et al.* 2011) and RM11-1a (Ruderfer *et al.* 2006) (Figure S9). Three of these amino acid changes (T313R, Q650E, N777T) are found in all *Saccharomyces cerevisiae* strains (except laboratory ones) and a fourth mutation (N475D) in all except 3 of the 39 strains sequenced at the Sanger Institute. Only the L559 was found in strains from different origins [*e.g*, wild (UWOPS87.2421, UWOPS05.217) or sake (Y12)]. The four common amino acids changes were also found in *ABZ1* orthologous of other *Saccharomyces* species, indicating that they correspond to an ancestral form of the gene. This situation is consistent with a divergence of the *ABZ1* gene of S288c. This evolution has been likely associated to a partial loss of function of the *p*-aminobenzoate synthase probably due to a cultivation of the strain in rich laboratory media (Kvitek *et al.* 2008). This idea is strengthened by additional information obtained from a large-scale analysis of fermentation phenotypes of *Saccharomyces* strains that included those from the Sanger list (Liti *et al.* 2009; Camarasa *et al.*; paper in preparation). It was observed that alteration of R_max_ in the absence of PABA was associated to Abz1 amino acids that are shared by laboratory strains (Camarasa, personal communication). This is consistent with an evolution *ABZ1* toward a defective form in the laboratory strains. The laboratory allele of *ABZ1* is probably not heavily defective since it does not lead to a true PABA auxotrophy. The strain S288c grows normally in a minimal medium without PABA with no change observed by comparison with a medium supplemented with 1 mg/l PABA) (data not shown). Such a situation may have facilitated the conservation of an *ABZ1* form that triggers a phenotype only under specific conditions.

More information based on variation of fermentation parameters and their relationships with expression will certainly be gained from the dissection of the QTL corresponding to the chromosome II hotspot that controls the fermentation rate in the late phase at R_70_ and consistently at R_50_. Several genes involved in stress responses (*HSP30*, *HSP12*, *SPS100*) whose level of expression correlated negatively with R_70_ have a linkage in the hotspot. These stress genes displayed a high level of expression in the laboratory strain and in the progeny that inherited the laboratory-strain form of the region (data not shown). This indicates that the locus from S288c leads to a specific stress response and a low fermentation rate. The potential mechanisms for this observation remain unknown. Our study did not address the expression of the genes acquired by the wine strain EC1118 from a horizontal transfer (Novo *et al.* 2009) as these genes were unknown at the beginning of the study. However, we have checked for linkage to makers flanking these regions and did not find any with fermentation traits and only one weak with an eQTL, suggesting that they have no strong impact on the parameters considered here.

The biological device used in the present study with a small population of segregants was able to detect fermentation QTL, but it had a lower power to detect eQTL. An additional difficulty for eQTL detection may originate from the specific physiological conditions under which we analyzed gene expression (*i.e.*, nongrowing cells under conditions of stress). Until now, yeast eQTL studies have been performed with growing cells. Under starvation and conditions of stress, many genes that respond to environmental changes have an expression more noisy than average (Gasch 2007; Razer *et al.* 2004). However, because most critical phenomena in industrial alcoholic fermentations (reduction in carbon flux, ethanol inhibition, cell death, *etc*.) take place during this phase, an assessment of the relationships between variations in gene expression and fermentation traits is required under such conditions. The findings of our study are the first step toward understanding this process. We demonstrated the role of a QTL relevant for alcoholic fermentation performance and provided an initial basis to address the relationships among fermentation QTL and variations in gene expression.

## Supplementary Material

Supporting Information
