# Supplementary material for: Deciphering the Molecular Basis of Wine Yeast Fermentation Traits Using a Combined Genetic and Genomic Approach
Source: G3 (Bethesda). 2011 Sep 1;1(4):263–81. doi: 10.1534/g3.111.000422 (PMC3276144; doi:10.1534/g3.111.000422)
Supplement: Supporting Information [file supp_1_4_263__index.html]

Supporting Information 

# Deciphering the Molecular Basis of Wine Yeast Fermentation Traits Using a Combined Genetic and Genomic Approach

## Supporting Information for Ambroset *et al.*, 2011

**Files in this Data Supplement:**

- Supporting Information - Figures S1-S9 and Tables S1-S4 (PDF, 2.5 MB)
- Figure S1 - Fermentation kinetics of EC1118 industrial strain (orange line), S288c laboratory strain (green curve), 59A (red curve), the hybrid Z59S (yellow curve) and some segregants (all other coloured curves) (PDF, 124 KB)
- Figure S2 - Distribution of fermentation parameters and metabolites production in the 30 segregants (PDF, 264 KB)
- Figure S3 - Fermentation traits value for each segregant and parental strains (PDF, 716 KB)
- Figure S4 - Correlations between fermentation parameters and the expression ratio of some genes in the population of segregants (PDF, 192 KB)
- Figure S5 - Microarray-derived marker map. Each vertical tick represents one genetic marker (PDF, 76 KB)
- Figure S6 - Markers distribution in the 30 segregants (PDF, 820 KB)
- Figure S7 - Overlapping of LOD peak values localised on chromosome II for four phenotypic traits (PDF, 60 KB)
- Figure S8 - Genomic distribution of eQTL. Positions of detected eQTL are plotted against the position of the regulated gene (PDF, 196 KB)
- Figure S9 - Alignment of the Abz1 amino acid modified in the strain 59A with the corresponding region seven other *Saccharomyces cerevisiae* strains and three other *Saccharomyces* species (PDF, 60 KB)
- Table S1 - Parental strains and segregants fermentation characteristics (PDF, 40 KB)
- Table S2 - Parental strains and segregants metabolites production characteristics (PDF, 40 KB)
- Table S3 - Genes showing strong differential expression between the two parental strains (PDF, 104 KB)
- Table S4 - Genes with eQTL in the 7 hotspots (PDF, 48 KB)
