# Supplementary material for: Deciphering the Molecular Basis of Wine Yeast Fermentation Traits Using a Combined Genetic and Genomic Approach
Source: G3 (Bethesda). 2011 Sep 1;1(4):263–81. doi: 10.1534/g3.111.000422 (PMC3276144; doi:10.1534/g3.111.000422)
Supplement: Supporting Information [file supp_1.4.263_000422SI.pdf]

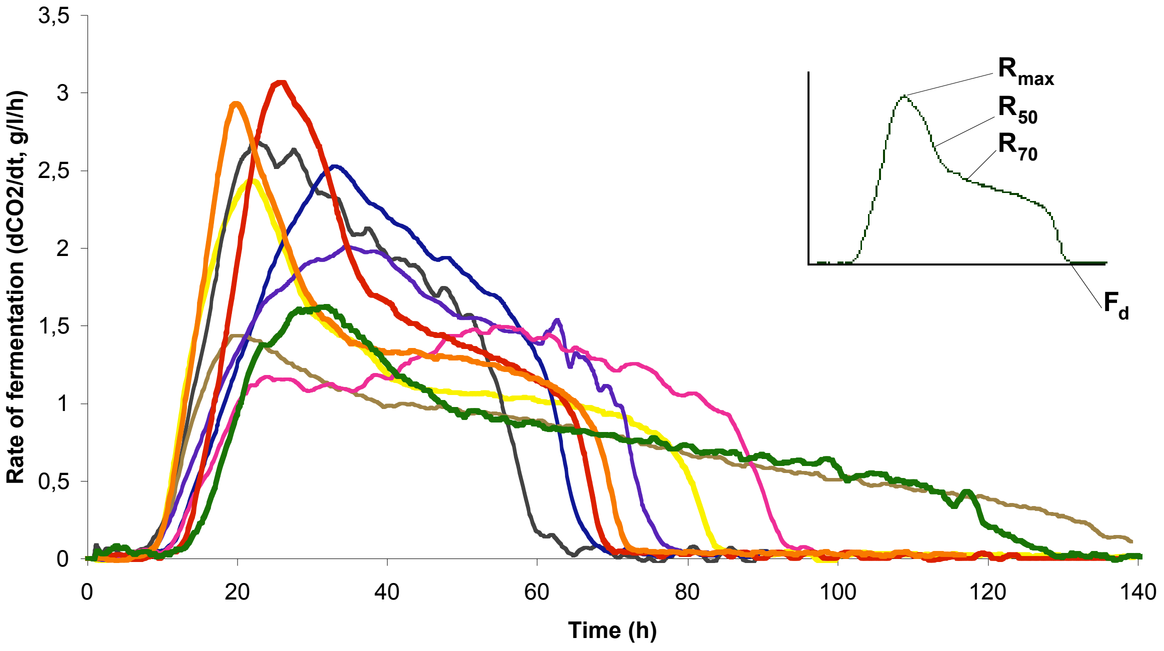

Figure S1 Fermentation kinetics of EC1118 industrial strain (orange line), S288c laboratory strain (green curve), 59A (red curve), the hybrid Z59S (yellow curve) and some segregants (all other coloured curves). The CO_2 production rate was measure in MS300 medium. ($g.L^{-1}.h^{-1}$).

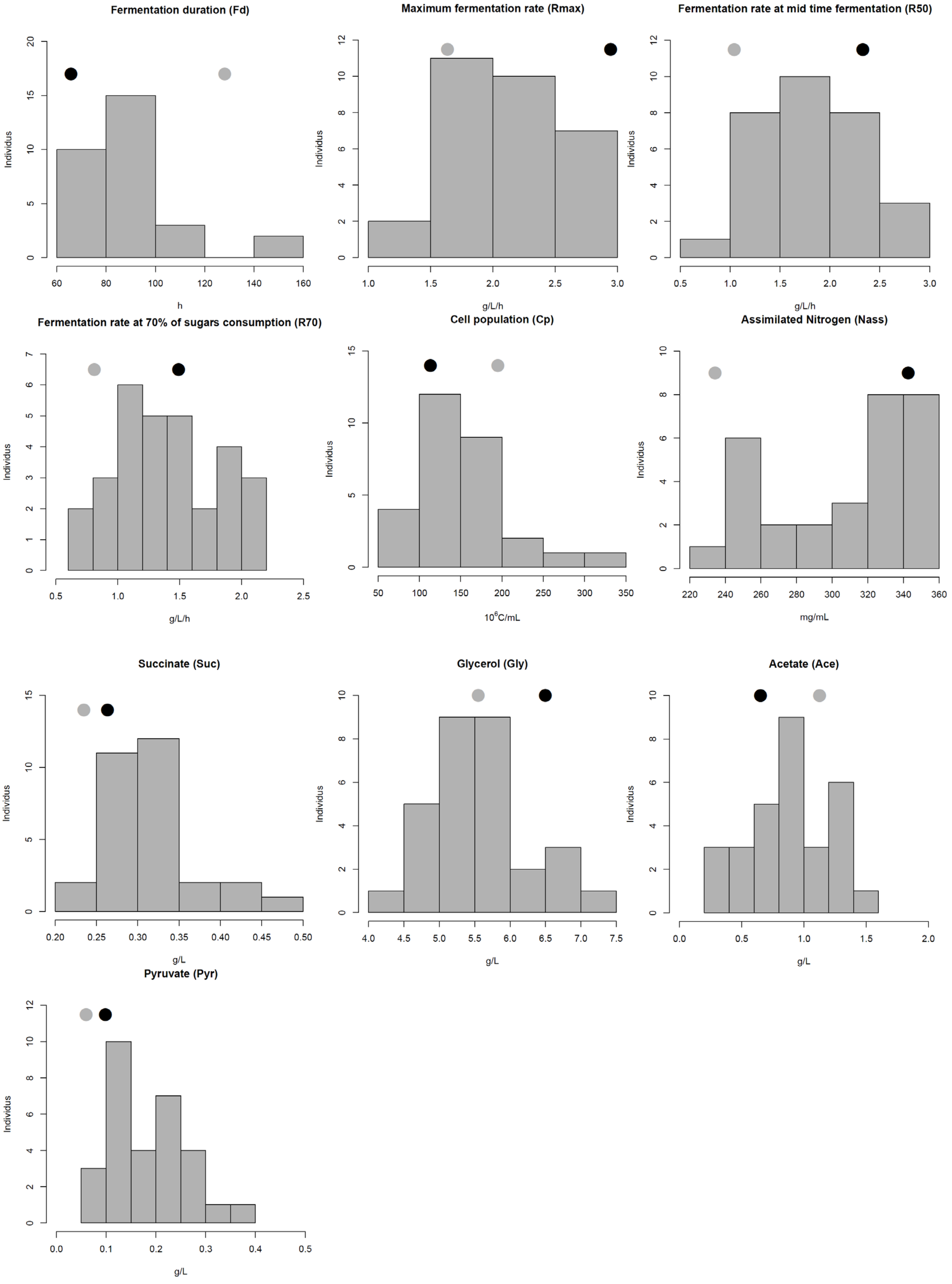

Figure S2 Distribution of fermentation parameters and metabolites production in the 30 segregants. Parent S288c and 59A mean values are shown on the top as grey (S288c) and black (59A) dots.

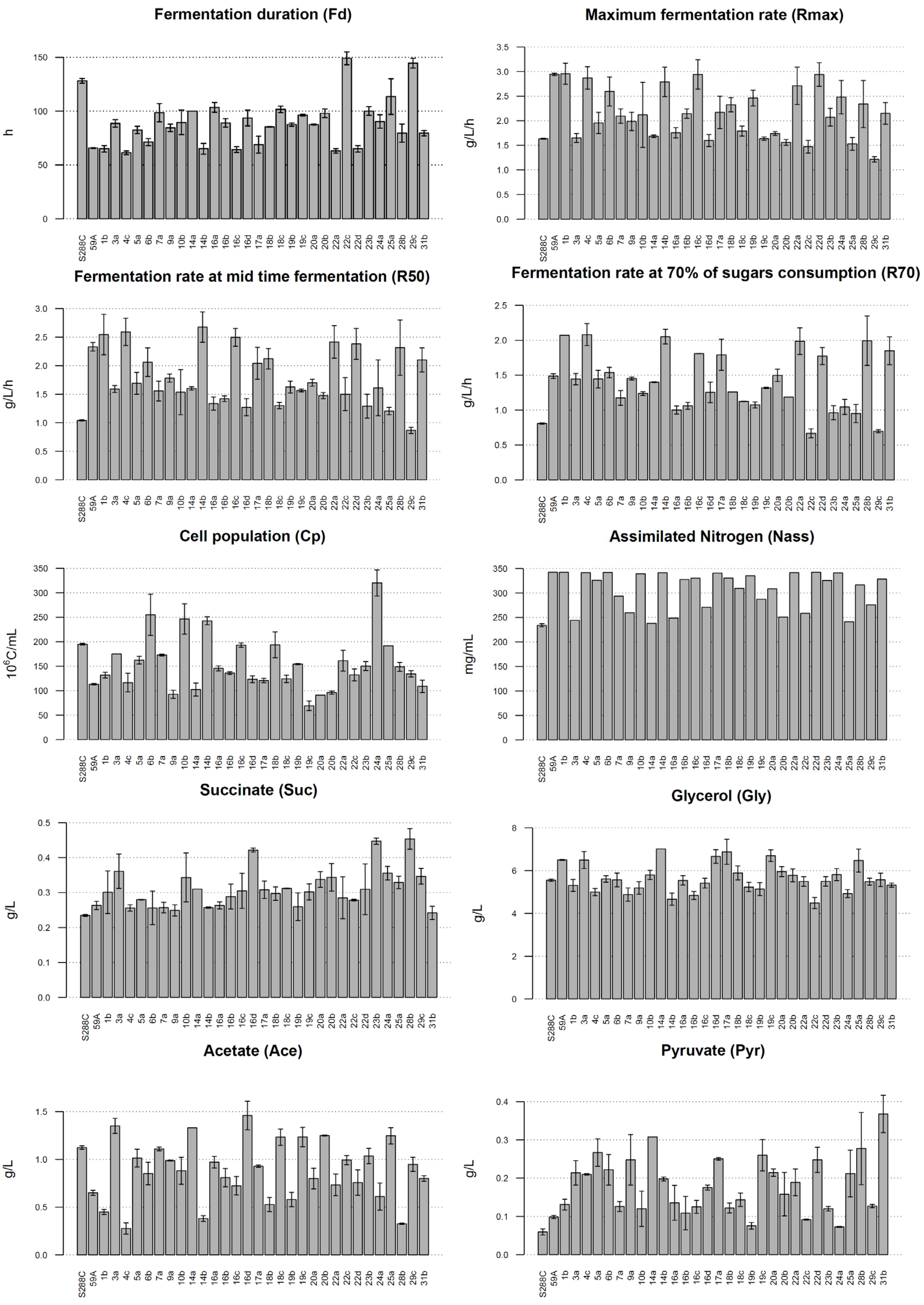

Figure S3 Fermentation traits value for each segregant and parental strains. The mean value and the standard deviation for each trait is provided. All traits were measured in two independent biological replicates except Nass measured from one fermentation experiment for the segregants and in five biological replicates for the parental strains and all traits.

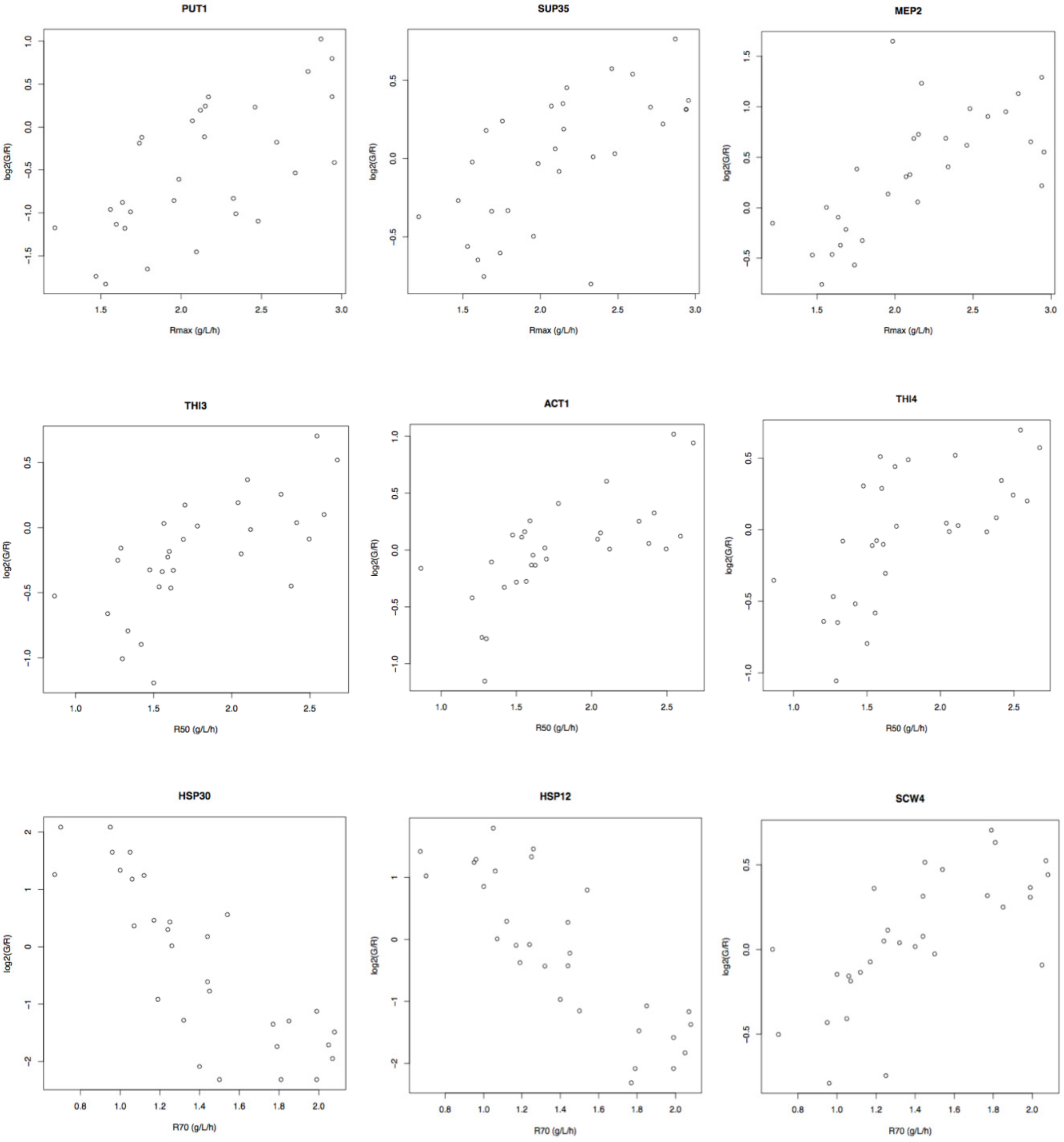

Figure S4 Correlations between fermentation parameters and the expression ratio of some genes in the population of segregants

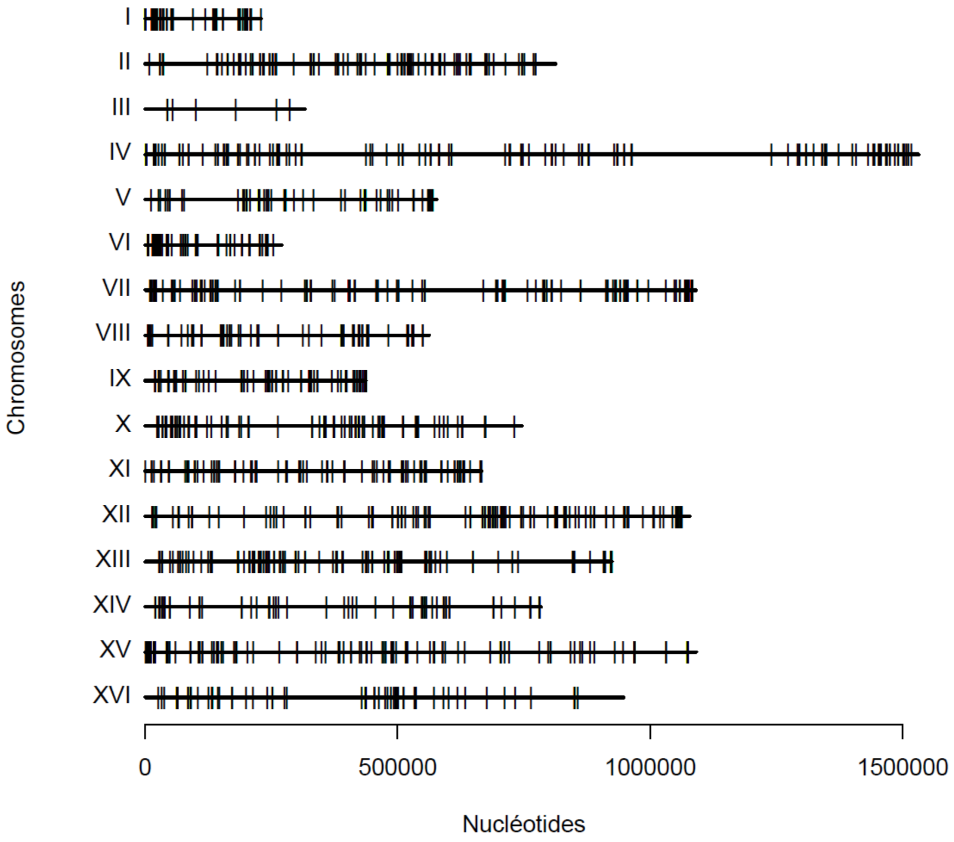

Figure S5 Microarray-derived marker map. Each vertical tick represents one genetic marker.

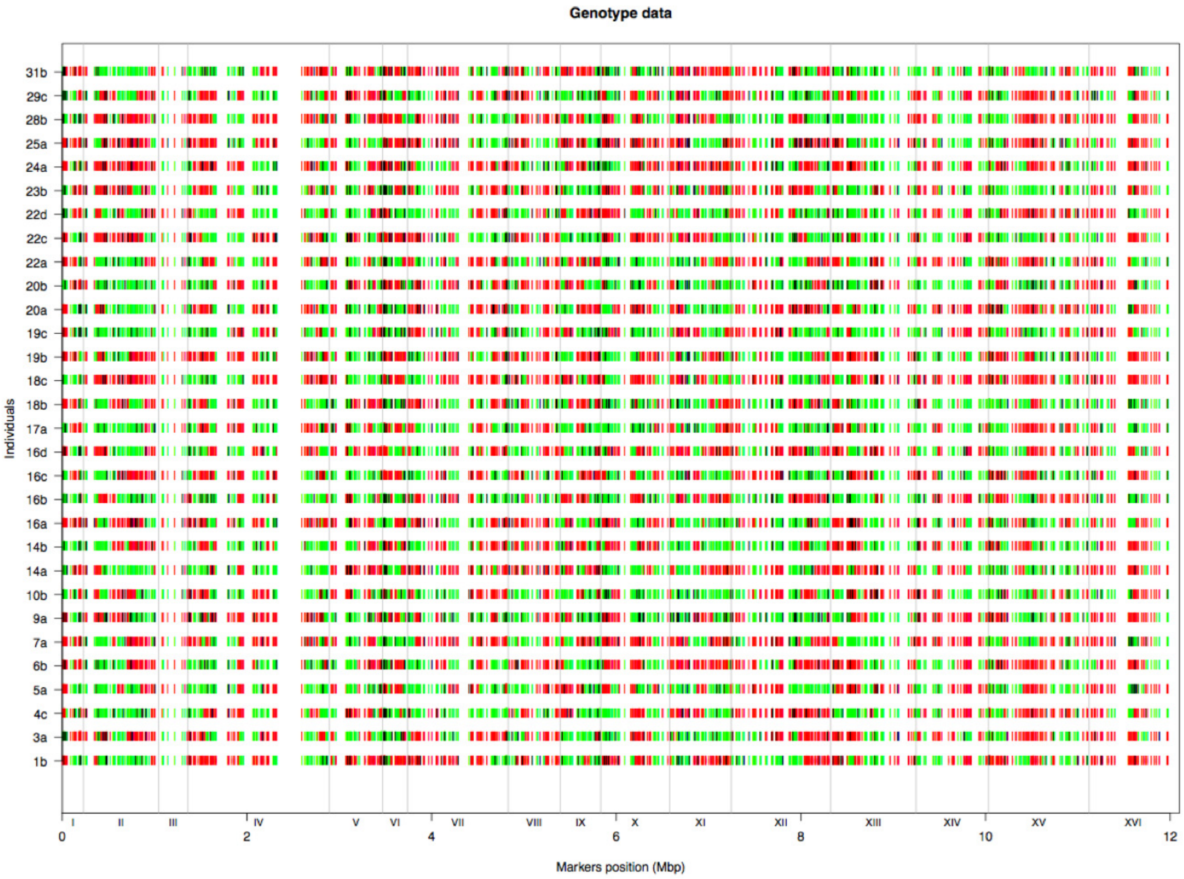

Figure S6 Markers distribution in the 30 segregants. Each marker position is displayed on the concatenated chromosomes with the colour indicating the parental origin (green 59A, red S288c). Ambiguous markers are indicated by a black bar.

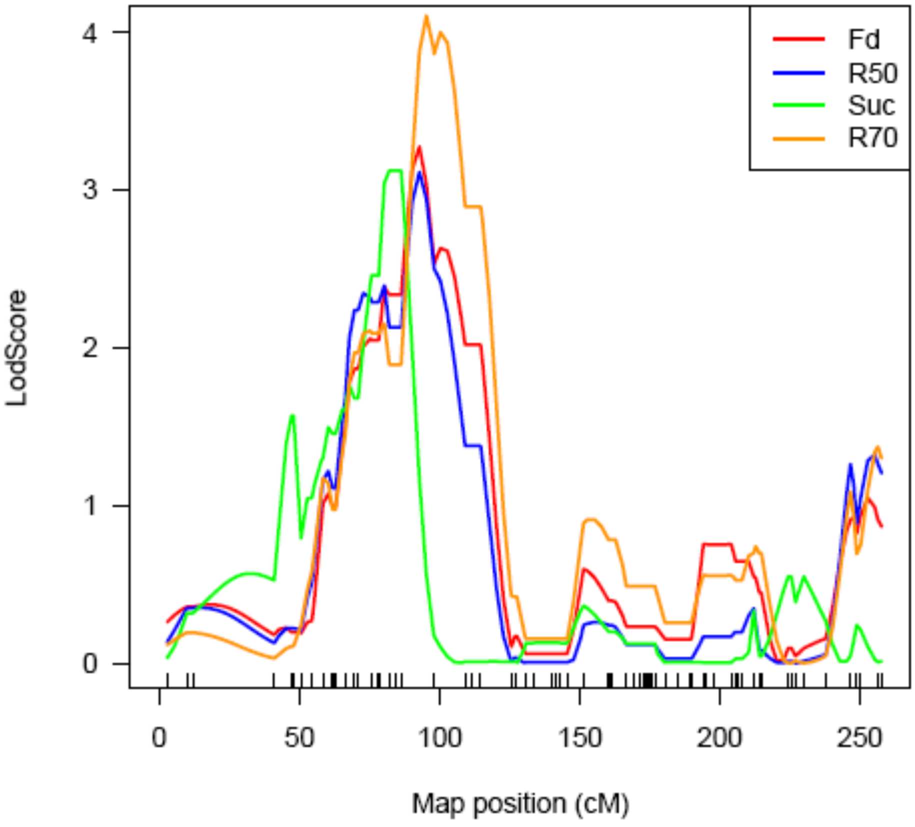

Figure S7 Overlapping of LOD peak values localised on chromosome II for four phenotypic traits. The x-axis represents the genomic marker localisation and the y-axis represents the LOD score values.

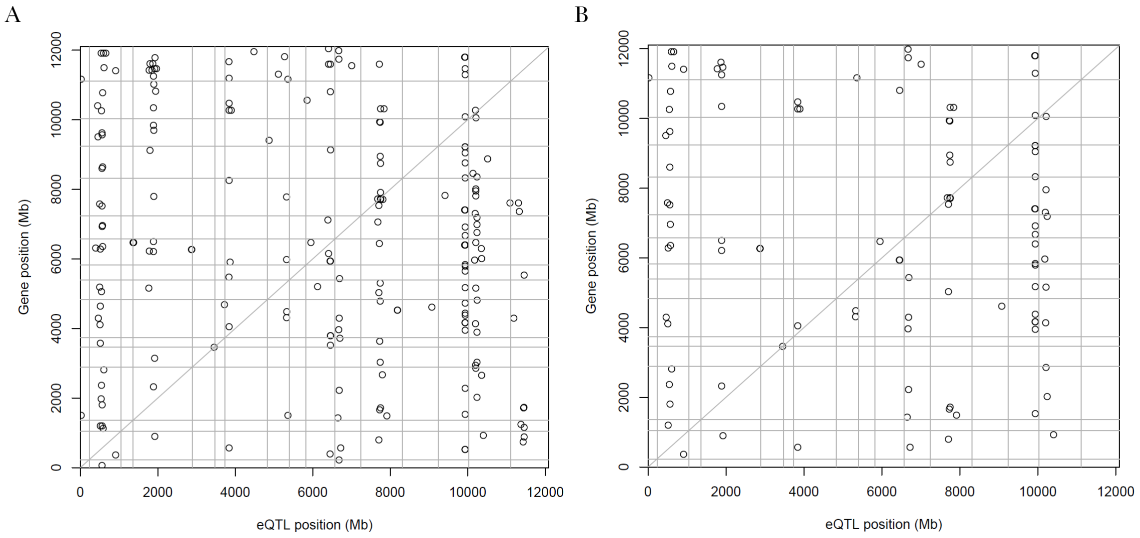

Figure S8 Genomic distribution of eQTL. Positions of detected eQTL are plotted against the position of the regulated gene. The figure displays eQTL with LOD score higher than 4.0 (A) and higher than 4.5 (B).

59 A	R	D	R	E	T
S. 288C	E N T G E	K K N I K	E F K D I	S V Q E Y	Y N K
RM11-1a	E N R G E	K K D I K	E F R D I	S V E E Y	Y T K
VIN13	E N R G E	K K D I K	E F R D I	S V E E Y	Y T K
YJM789	E N R G E	K K D I K	E F R D I	S V E E Y	Y T K
Y12	E N R G E	K K D I K	E F K D I	S V E E Y	Y T K
UWOPS87.2421	E N R G E	K K D I K	E F K D I	S V E E Y	Y T K
UWOPS05.217	E N R G E	K K D I K	E F K D I	S V E E Y	Y T K
S .bayanus	E N R G E	K K D V E	E F N D I	S V E E Y	Y T K
S. mikatae	E N R G E	K K D V R	E F N D I	S V E E Y	Y T K
S. paradoxus	E N R G E	K E D V R	E F N D I	S V E E Y	Y T K
	313	475	559	650	777

Figure S9 Alignment of the Abz1 amino acid modified in the strain 59A with the corresponding region seven other *Saccharomyces cerevisiae* strains and three other *Saccharomyces* species.

Table S1   Parental strains and segregants fermentation characteristics

Strains	Rmax	R50	R70	Fd	Cp	Nass
S288C	1.63 (0.03)	1.04 (0.02)	0.81 (0.02)	128 (4.8)	194.9 (3.6)	234 (7)
59A	2.94 (0.05)	2.33 (0.17)	1.49 (0.08)	66 (0.7)	113.2 (3.5)	343 (0)
1b	2.96 (0.3)	2.54 (0.5)	2.07 (NA)	65 (4.2)	131.8 (8.3)	343 (NA)
3a	1.65 (0.13)	1.59 (0.08)	1.44 (0.12)	88 (4.9)	175 (0)	244 (NA)
4c	2.87 (0.33)	2.59 (0.34)	2.08 (0.22)	61 (2.5)	116.5 (26.9)	342 (NA)
5a	1.96 (0.3)	1.69 (0.27)	1.44 (0.18)	82 (4.9)	162.2 (11)	326 (NA)
6b	2.59 (0.42)	2.06 (0.35)	1.54 (0.13)	71 (4.6)	255 (59.4)	342 (NA)
7a	2.1 (0.21)	1.55 (0.25)	1.17 (0.15)	98 (12)	172.9 (2.7)	294 (NA)
9a	1.98 (0.26)	1.78 (0.1)	1.45 (0.03)	84 (4.9)	92.7 (11.7)	260 (NA)
10b	2.12 (0.93)	1.53 (0.56)	1.24 (0.04)	90 (16.3)	246.3 (43.7)	339 (NA)
14a	1.68 (0.04)	1.6 (0.04)	1.4 (0.01)	100 (0)	102.3 (18.8)	238 (NA)
14b	2.79 (0.42)	2.67 (0.37)	2.05 (0.15)	65 (7.1)	242.6 (11.5)	342 (NA)
16a	1.75 (0.15)	1.34 (0.16)	1 (0.08)	104 (6.4)	145.4 (7.3)	249 (NA)
16b	2.15 (0.13)	1.42 (0.07)	1.06 (0.07)	89 (5.7)	135.9 (4)	328 (NA)
16c	2.94 (0.42)	2.5 (0.22)	1.81 (0)	64 (3.9)	192.9 (6.2)	330 (NA)
16d	1.6 (0.21)	1.27 (0.26)	1.25 (0.21)	94 (10.5)	123.3 (9.6)	271 (NA)
17a	2.17 (0.47)	2.04 (0.4)	1.79 (0.32)	69 (11)	120.8 (6)	341 (NA)
18b	2.33 (0.21)	2.12 (0.25)	1.26 (NA)	85 (0.4)	193.5 (37.5)	330 (NA)
18c	1.79 (0.17)	1.3 (0.1)	1.12 (0)	102 (5.3)	124.1 (10.6)	309 (NA)
19b	2.46 (0.23)	1.62 (0.15)	1.07 (0.06)	88 (2.1)	154.3 (1.5)	335 (NA)
19c	1.64 (0.05)	1.56 (0.04)	1.32 (0.01)	96 (1.1)	69 (13.8)	287 (NA)
20a	1.74 (0.06)	1.7 (0.08)	1.5 (0.12)	88 (0.7)	90.6 (NA)	308 (NA)
20b	1.56 (0.08)	1.48 (0.08)	1.19 (NA)	98 (5.7)	95.8 (4.5)	251 (NA)
22a	2.71 (0.54)	2.42 (0.4)	1.99 (0.27)	63 (2.8)	161.1 (29.8)	342 (NA)
22c	1.47 (0.18)	1.5 (0.41)	0.67 (0.09)	149 (8.5)	132.2 (17.3)	258 (NA)
22d	2.94 (0.34)	2.38 (0.38)	1.77 (0.17)	65 (4.2)	NaN (NA)	343 (NA)
23b	2.07 (0.25)	1.29 (0.3)	0.96 (0.14)	100 (5.7)	150.2 (12.9)	326 (NA)
24a	2.48 (0.48)	1.61 (0.69)	1.05 (0.15)	90 (9)	319.9 (37.6)	341 (NA)
25a	1.53 (0.18)	1.21 (0.09)	0.95 (0.18)	114 (23.3)	191.6 (NA)	241 (NA)
28b	2.34 (0.68)	2.31 (0.69)	1.99 (0.5)	80 (12)	148.6 (12.8)	316 (NA)
29c	1.21 (0.08)	0.86 (0.08)	0.7 (0.03)	144 (6.4)	134.4 (8.9)	276 (NA)
31b	2.15 (0.31)	2.1 (0.3)	1.85 (0.28)	80 (3.5)	108.8 (18)	328 (NA)

Standard deviation for each trait is indicated between parentheses.

Table S2 Parental strains and segregants metabolites production characteristics

Strains	Suc	Gly	Ace	Pyr
S288C	0.23 (0.01)	5.55 (0.12)	1.12 (0.04)	0.06 (0.02)
59A	0.26 (0.03)	6.5 (0.06)	0.65 (0.06)	0.1 (0.01)
1b	0.3 (0.09)	5.31 (0.4)	0.45 (0.04)	0.13 (0.02)
3a	0.36 (0.07)	6.49 (0.56)	1.35 (0.11)	0.21 (0.05)
4c	0.26 (0.01)	5 (0.24)	0.28 (0.08)	0.21 (0)
5a	0.28 (0)	5.61 (0.21)	1.01 (0.13)	0.27 (0.05)
6b	0.26 (0.07)	5.56 (0.45)	0.85 (0.17)	0.22 (0.06)
7a	0.26 (0.02)	4.88 (0.44)	1.11 (0.03)	0.13 (0.02)
9a	0.25 (0.02)	5.19 (0.42)	0.99 (0.01)	0.25 (0.09)
10b	0.34 (0.1)	5.8 (0.3)	0.88 (0.2)	0.12 (0.07)
14a	0.31 (NA)	7.01 (NA)	1.33 (NA)	0.31 (NA)
14b	0.26 (0)	4.66 (0.4)	0.38 (0.04)	0.2 (0.01)
16a	0.26 (0.01)	5.54 (0.31)	0.97 (0.09)	0.14 (0.06)
16b	0.29 (0.05)	4.84 (0.26)	0.81 (0.14)	0.11 (0.06)
16c	0.3 (0.07)	5.41 (0.32)	0.72 (0.14)	0.12 (0.02)
16d	0.42 (0.01)	6.66 (0.44)	1.46 (0.21)	0.18 (0.01)
17a	0.31 (0.04)	6.88 (0.82)	0.93 (0.02)	0.25 (0)
18b	0.3 (0.03)	5.89 (0.47)	0.53 (0.1)	0.12 (0.02)
18c	0.31 (0)	5.23 (0.3)	1.23 (0.12)	0.14 (0.02)
19b	0.26 (0.06)	5.13 (0.43)	0.58 (0.11)	0.08 (0.01)
19c	0.3 (0.03)	6.7 (0.38)	1.23 (0.14)	0.26 (0.06)
20a	0.34 (0.03)	5.95 (0.33)	0.8 (0.15)	0.21 (0.01)
20b	0.34 (0.06)	5.78 (0.43)	1.25 (0.01)	0.16 (0.08)
22a	0.28 (0.08)	5.49 (0.32)	0.73 (0.16)	0.19 (0.05)
22c	0.28 (0)	4.48 (0.37)	0.99 (0.07)	0.09 (0)
22d	0.31 (0.1)	5.5 (0.32)	0.76 (0.19)	0.25 (0.05)
23b	0.45 (0.01)	5.81 (0.4)	1.03 (0.11)	0.12 (0.01)
24a	0.36 (0.03)	4.93 (0.26)	0.61 (0.2)	0.07 (0)
25a	0.33 (0.03)	6.47 (0.76)	1.25 (0.12)	0.21 (0.09)
28b	0.45 (0.04)	5.48 (0.23)	0.33 (0.01)	0.28 (0.13)
29c	0.35 (0.03)	5.57 (0.44)	0.95 (0.1)	0.13 (0.01)
31b	0.24 (0.03)	5.32 (0.13)	0.8 (0.04)	0.37 (0.07)

Standard deviation for each trait is indicated between parentheses.

Table S3 Genes showing strong differential expression between the two parental strains. (A) Genes overexpressed in 59A. (B) Genes overexpressed in S288c.

A

Name	logFC	Ratio	Adj PVal	Function
PAU5	3.256	9.555	8.56E-16	Part of 23-member seripauperin multigene family encoded mainly in subtelomeric regions
PAU4	3.242	9.458	9.26E-15	Part of 23-member seripauperin multigene family encoded mainly in subtelomeric regions
PAU12	2.83	7.112	1.97E-15	Hypothetical protein
PAU20	2.827	7.094	3.61E-15	Hypothetical protein
PAU6	2.483	5.592	1.43E-13	Part of 23-member seripauperin multigene family encoded mainly in subtelomeric regions
PAU1	2.412	5.322	1.78E-14	Part of 23-member seripauperin multigene family encoded mainly in subtelomeric regions
PAU13	2.324	5.007	8.02E-13	Putative protein of unknown function
INO1	2.287	4.88	1.05E-12	Inositol 1-phosphate synthase
PAU10	2.281	4.861	7.24E-14	Hypothetical protein
PAU14	2.239	4.721	7.27E-14	Hypothetical protein
PAU11	2.214	4.64	7.88E-13	Putative protein of unknown function
PAU8	2.133	4.385	7.85E-12	Hypothetical protein
PAU7	2.038	4.107	3.78E-14	Part of 23-member seripauperin multigene family
PAU18	1.973	3.926	1.96E-12	Hypothetical protein
HXK1	1.877	3.674	2.70E-10	Hexokinase isoenzyme 1
PAU19	1.85	3.606	3.87E-13	Hypothetical protein
HUG1	1.752	3.368	1.51E-11	Protein involved in the Mec1p-mediated checkpoint pathway that responds to DNA damage or replication arrest
TRK2	1.642	3.122	2.47E-11	Component of the Trk1p-Trk2p potassium transport system
YGR126W	1.641	3.119	3.61E-11	Putative protein of unknown function
YNL324W	1.638	3.112	2.74E-12	Dubious ORF unlikely to encode a functional protein
YDR102C	1.589	3.008	4.15E-12	Dubious open reading frame
YPR126C	1.558	2.945	2.35E-14	Dubious open reading frame unlikely to encode a functional protein
DAN3	1.549	2.925	5.89E-10	Cell wall mannoprotein with similarity to Tir1p
DDR48	1.544	2.917	1.37E-07	DNA damage-responsive protein
DAN2	1.513	2.855	1.23E-12	Cell wall mannoprotein with similarity to Tir1p
GPH1	1.437	2.707	1.05E-12	Non-essential glycogen phosphorylase required for the mobilization of glycogen
MFA2	1.413	2.664	4.15E-09	Mating pheromone a-factor
YDL085C-A	1.402	2.643	8.24E-09	Putative protein of unknown function
YPR050C	1.373	2.59	1.28E-12	Dubious open reading frame unlikely to encode a protein
YDR355C	1.334	2.521	9.76E-12	Dubious: Dubious open reading frame unlikely to encode a protein
YIL067C	1.326	2.507	5.74E-12	Uncharacterized protein of unknown function
YCL020W	1.302	2.466	2.57E-09	Retrotransposon TYA Gag gene co-transcribed with TYB Pol
SOL2	1.301	2.464	9.67E-11	Protein with a possible role in tRNA export
JSN1	1.272	2.415	9.71E-14	Member of the Puf family of RNA-binding proteins
GPM1	1.271	2.412	7.16E-10	Tetrameric phosphoglycerate mutase
MSG5	1.256	2.389	4.09E-10	Dual-specificity protein phosphatase required for maintenance of a low level of signaling through the cell integrity pathway
DBP5	1.248	2.375	3.52E-10	Cytoplasmic ATP-dependent RNA helicase of the DEAD-box family involved in mRNA export from the nucleus
YBR099C	1.246	2.372	4.78E-11	Dubious open reading frame unlikely to encode a protein
SDS24	1.223	2.334	1.77E-10	One of two S
HRT1	1.221	2.33	1.20E-11	RING finger containing subunit of Skp1-Cullin-F-box ubiquitin protein ligases (SCF)
PAU17	1.218	2.326	2.77E-09	Putative protein of unknown function
PEX11	1.207	2.309	8.92E-11	Peroxisomal membrane protein required for peroxisome proliferation and medium-chain fatty acid oxidation

MSH6	1.206	2.307	4.89E-09	Protein required for mismatch repair in mitosis and meiosis
FLO5	1.205	2.306	1.71E-13	Lectin-like cell wall protein (flocculin) involved in flocculation
YFL052W	1.205	2.306	1.93E-13	Putative zinc cluster protein that contains a DNA binding domain
YKR045C	1.202	2.3	2.57E-10	Putative protein of unknown function
YJL127W-A	1.201	2.298	1.22E-08	Dubious open reading frame unlikely to encode a protein
DAN1	1.2	2.297	3.92E-10	Cell wall mannoprotein with similarity to Tir1p
ENP1	1.189	2.28	2.20E-08	Protein associated with U3 and U14 snoRNAs
YHR049C-A	1.188	2.279	7.03E-11	Dubious open reading frame unlikely to encode a functional protein

B

Name	logFC	Ratio	Adj PVal	Function
PLC1	4.693	25.864	4.07E-19	Phospholipase C
YNL284C-A*	4.252	19.047	1.89E-19	Retrotransposon TYA Gag gene co-transcribed with TYB Pol
AI2*	4.235	18.833	3.92E-18	Reverse transcriptase required for splicing of the COX1 pre-mRNA
RTC4	3.439	10.844	3.68E-18	Putative protein of unknown function
CDC5	2.857	7.246	2.82E-17	Polo-like kinase with similarity to Xenopus Plx1 and S
ARH1	2.77	6.823	1.55E-17	Oxidoreductase of the mitochondrial inner membrane
YGR051C	2.53	5.776	2.24E-17	Dubious open reading frame unlikely to encode a protein
YER084W	2.49	5.617	4.91E-16	Dubious open reading frame unlikely to encode a protein
YNL285W	2.459	5.498	1.12E-17	Dubious open reading frame unlikely to encode a functional protein
ELG1	2.437	5.413	1.57E-14	Protein required for S phase progression and telomere homeostasis
ATE1	2.41	5.316	1.89E-19	Arginyl-tRNA-protein transferase
YER138W-A	2.379	5.201	5.74E-12	Putative protein of unknown function
SCH9	2.365	5.152	3.30E-16	Protein kinase involved in transcriptional activation of osmostress-responsive genes
YBL107W-A	2.234	4.705	6.28E-14	Dubious open reading frame unlikely to encode a protein
RSA1	2.204	4.609	5.55E-18	Protein involved in the assembly of 60S ribosomal subunits
THI80	2.157	4.459	1.55E-16	Thiamine pyrophosphokinase
HSP30	2.121	4.349	3.76E-08	Hydrophobic plasma membrane localized
YBL111C	2.068	4.192	9.79E-18	Helicase-like protein encoded within the telomeric Y element
YHR218W	2.018	4.051	2.21E-16	Helicase-like protein encoded within the telomeric Y element
YIR014W	1.93	3.809	2.68E-15	Putative protein of unknown function
GIN4	1.894	3.717	1.51E-17	Protein kinase involved in bud growth and assembly of the septin ring
HSP12	1.885	3.695	2.94E-14	Plasma membrane localized protein that protects membranes from desiccation
PBP1	1.857	3.623	1.05E-14	Protein interacting with poly(A)-binding protein Pab1p
YHL005C	1.842	3.584	6.15E-17	Dubious open reading frame unlikely to encode a protein
AUA1	1.801	3.485	8.96E-09	Protein required for the negative regulation by ammonia of Gap1p
TPO3	1.8	3.481	9.65E-14	Polyamine transport protein specific for spermine
RPL2B	1.767	3.402	2.51E-14	Protein component of the large (60S) ribosomal subunit
YER053C-A	1.745	3.352	1.89E-06	Putative protein of unknown function
MCM22	1.737	3.334	6.76E-15	Protein involved in minichromosome maintenance
ASP3-1	1.734	3.326	9.66E-15	Cell-wall L-asparaginase II
SPS100	1.712	3.276	1.94E-11	Protein required for spore wall maturation
ASP3-3	1.704	3.257	4.88E-14	Cell-wall L-asparaginase II
NPY1	1.669	3.18	5.89E-15	NADH diphosphatase (pyrophosphatase)
HST1	1.665	3.172	3.56E-15	NAD(+)-dependent histone deacetylase
YLR281C	1.665	3.17	1.01E-16	Putative protein of unknown function
SPR3	1.644	3.125	3.61E-15	Sporulation-specific homolog of the yeast CDC3/10/11/12 family of bud neck microfilament genes
ASP3-2	1.628	3.09	1.70E-15	Cell-wall L-asparaginase II
YNL260C	1.614	3.06	1.72E-15	Putative protein of unknown function with similarity to a human protein overexpressed in oral cancers
KAR9	1.581	2.991	8.55E-10	Karyogamy protein required for correct positioning of the mitotic spindle and for orienting cytoplasmic microtubules
VIP1	1.577	2.984	8.36E-14	Inositol hexakisphosphate (IP6) and inositol heptakisphosphate (IP7) kinase

DOT1	1.572	2.973	1.32E-13	Nucleosomal histone H3-Lys79 methylase
AI3	1.557	2.942	1.99E-13	Endonuclease I-SceIII
MRPL13	1.54	2.909	6.20E-13	Mitochondrial ribosomal protein of the large subunit
YLR280C	1.54	2.909	5.89E-15	Dubious open reading frame unlikely to encode a functional protein
ASP3-4	1.536	2.899	2.40E-15	Cell-wall L-asparaginase II
ITC1	1.519	2.865	6.51E-14	Component of the ATP-dependent Isw2p-Itc1p chromatin remodeling complex
YIL089W	1.511	2.851	6.00E-13	Putative protein of unknown function
RPA49	1.498	2.824	7.21E-06	RNA polymerase I subunit A49
HXT5	-1.477	2.783	3.80 E-15	Hexose transporter
THI72	-1,477	2.783	1.71 E-11	Transporter of thiamine or related compound

For each gene the log_2 of fold change, the ratio of expression, the adjusted p-value and the function are indicated.
These are Ty1 genes, only one member of each was considered since they cross hybridize.

Table S4 Genes with eQTL in the 7 hotspots. Hotspot lod > 3.5

Hotspot	Gene				
**Chr II**	YAL044W-A	SSZ1	YMR031C	PMP3	MNE1
**87.8 - 125.3 cM**	FLC2	SPS100	NRK1	HSP12	ISA2
**263400 – 3759000 bp**	TDP1	CYR1	RPL42A	FMP37	PMT6
	HSP30	HXT8	COQ10	YGR079W	ECM12
	NTH1	SPC1	GSH2	CYS4	GCN3
	YDR053W	OSM1	DDR2	PTM1	EMP46
	TVP23	GPX1	MCT1	YKL165C-A	YMR010W
**Chr IV**	SFT2	STR2	YOR352W	YJL028W	SWR1
**161 – 188.2 cM**	DAD1	PNP1	SMA1	ISY1	RHO2
**483000 – 564600 bp**	YDR249C	YNL019C	ELP4	IZH2	FMP40
	YPL264C				
**Chr XI**	YAR069C	TIM21	YLR104W	YFR016C	SEC4
**29.2 – 45.5**	MRPL11	THI4	SIP3	FRS1	
**87600 – 136500 bp**	UME6	POT1	CSR2	ORC4	
**Chr XII**	CWH43	RPL43B	CTF18	SHE10	YPL034W
**147.7 – 171.6 cM**	MCH1	ASP3-1	SSO2	YOR1	GSM1
**443100 – 514800 bp**	YDL086W	ASP3-2	NGL2	IKI1	
	YEL008W	ASP3-3	HUB1	VPS63	
	IOC3	ASP3-4	MRPS12	YUH1	
**Chr XIV**	CDC15	HAM1	MTF1	VOA1	DDC1
**211.6 - 244 cM**	OLA1	MOG1	YMR262W	ENP2	JID1
**634800 – 732000 bp**	SAS10	YKL050C	DIA1	YGR251W	YIL055C
	DHH1	MTR2	ATG4	YRF1-3	RPS24B
	PCF11	SPA2	RRP40	LSM12	MDE1
	PNC1	YLR012C	YOR021C	YML131W	YJL225C
	NSA1	GAT3	RPL3	YMR086W	
	PEX14	RFX1	YOR289W	MRS1	
	PIL1	YLR243W	YPL107W	KAR2	
**Chr XV**	PGS1	CDC12	CHS5	SWC4	YOL047C
**51.4 - 71.4 cM**	ESF1	KRE27	RSC2	YGR265W	YOR050C
**154200 – 214200 bp**	MUS81	SNX4	ATG17	YHR003C	GCY1
	YDR532C	EXO70	OGG1	YLR257W	DSS4
	AFG1	NNF1	ALO1	MEC3	FRE1
	MST27	MLP1	ILV2	SFH1	VPS75
	HAP2				
**Chr XVI**	ATG12	MRP10	YEL076C	IDP3	
**96 – 126 cM**	YCP4	MCH1	ROK1	YEL008W	
**288000 – 378000 bp**	BUD23	CHL4	HIS5	YCR101C	

Genes which have a LOD score higher than 3.5 in the hotspot are displayed.
