# Supplementary material for: Deciphering the Molecular Basis of Wine Yeast Fermentation Traits Using a Combined Genetic and Genomic Approach
Source: G3 (Bethesda). 2011 Sep 1;1(4):263–81. doi: 10.1534/g3.111.000422 (PMC3276144; doi:10.1534/g3.111.000422)
Supplement: Supporting Information [file supp_1.4.263_FigureS1.pdf]

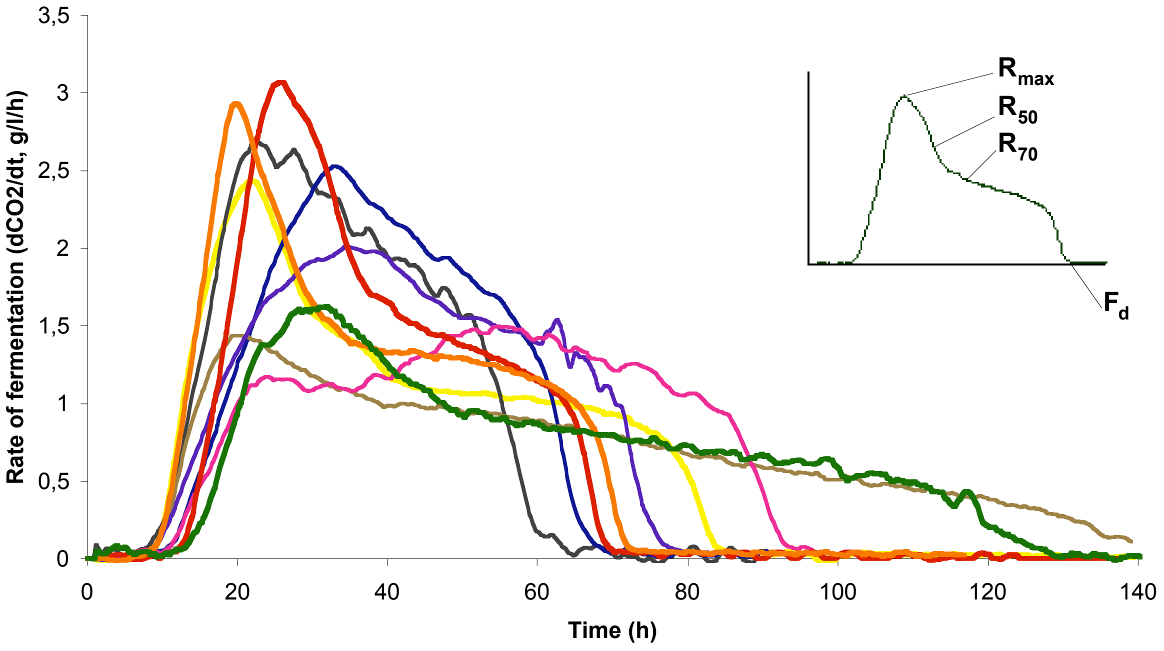

Figure S1 Fermentation kinetics of EC1118 industrial strain (orange line), S288c laboratory strain (green curve), 59A (red curve), the hybrid Z59S (yellow curve) and some segregants (all other coloured curves). The CO_2 production rate was measure in MS300 medium. ($g.L^{-1}.h^{-1}$).
