# Supplementary material for: Deciphering the Molecular Basis of Wine Yeast Fermentation Traits Using a Combined Genetic and Genomic Approach
Source: G3 (Bethesda). 2011 Sep 1;1(4):263–81. doi: 10.1534/g3.111.000422 (PMC3276144; doi:10.1534/g3.111.000422)
Supplement: Supporting Information [file supp_1.4.263_FigureS2.pdf]

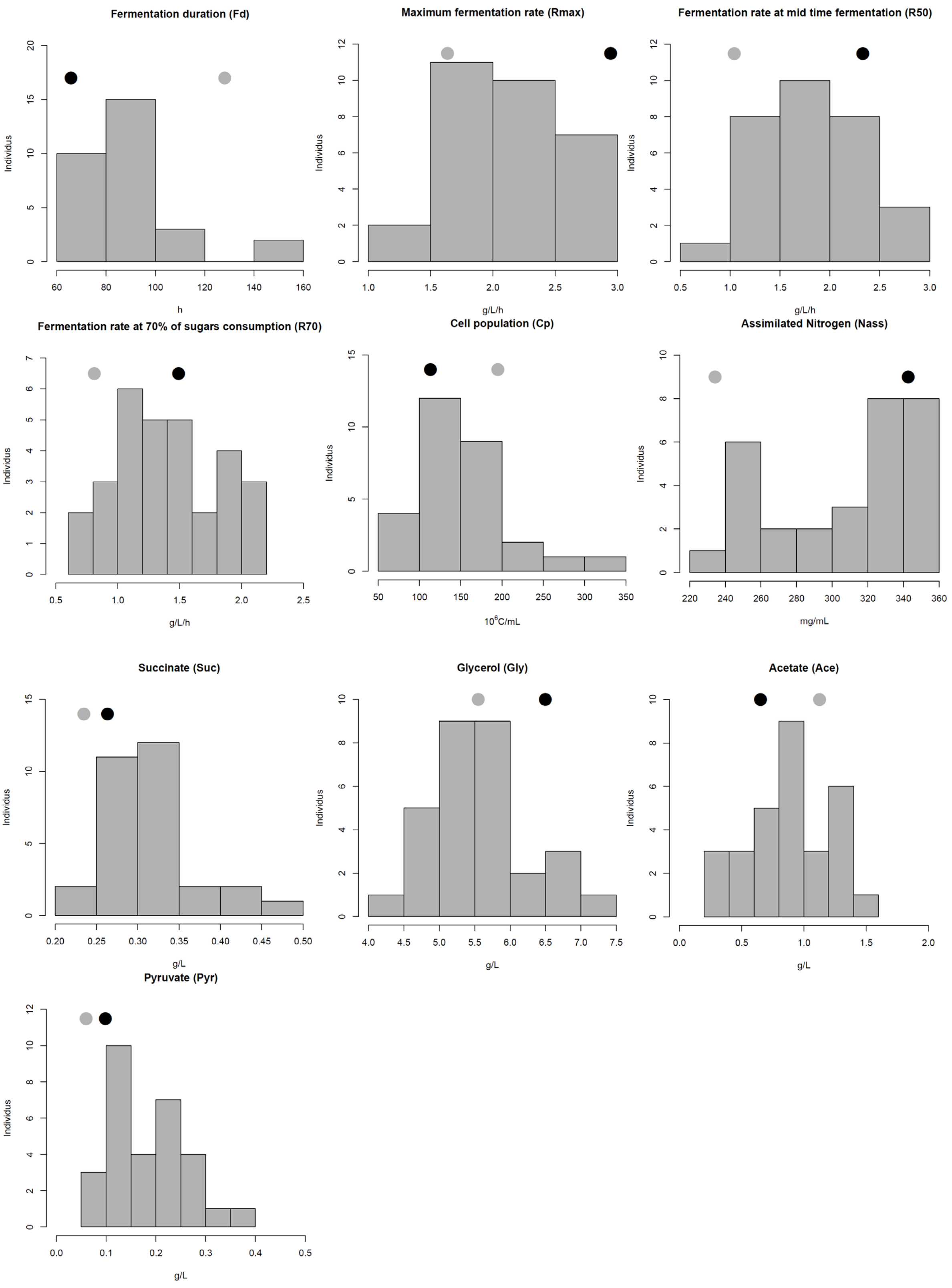

Figure S2 Distribution of fermentation parameters and metabolites production in the 30 segregants. Parent S288c and 59A mean values are shown on the top as grey (S288c) and black (59A) dots.
