# Supplementary material for: Deciphering the Molecular Basis of Wine Yeast Fermentation Traits Using a Combined Genetic and Genomic Approach
Source: G3 (Bethesda). 2011 Sep 1;1(4):263–81. doi: 10.1534/g3.111.000422 (PMC3276144; doi:10.1534/g3.111.000422)
Supplement: Supporting Information [file supp_1.4.263_FigureS3.pdf]

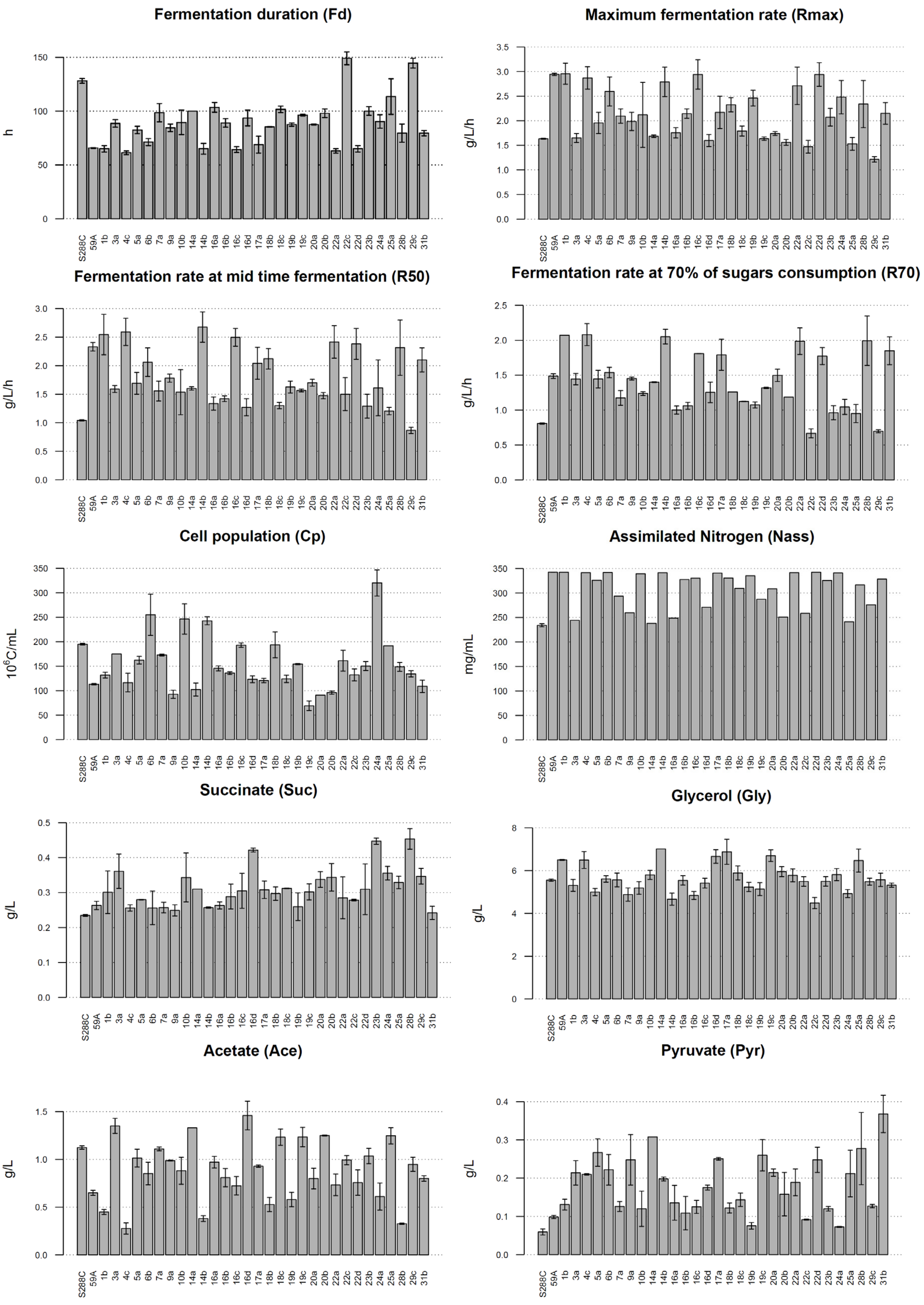

Figure S3 Fermentation traits value for each segregant and parental strains. The mean value and the standard deviation for each trait is provided. All traits were measured in two independent biological replicates except Nass measured from one fermentation experiment for the segregants and in five biological replicates for the parental strains and all traits.
