# Supplementary figures and images for: Deciphering the Molecular Basis of Wine Yeast Fermentation Traits Using a Combined Genetic and Genomic Approach
Source: G3 (Bethesda). 2011 Sep 1;1(4):263–81. doi: 10.1534/g3.111.000422 (PMC3276144; doi:10.1534/g3.111.000422)

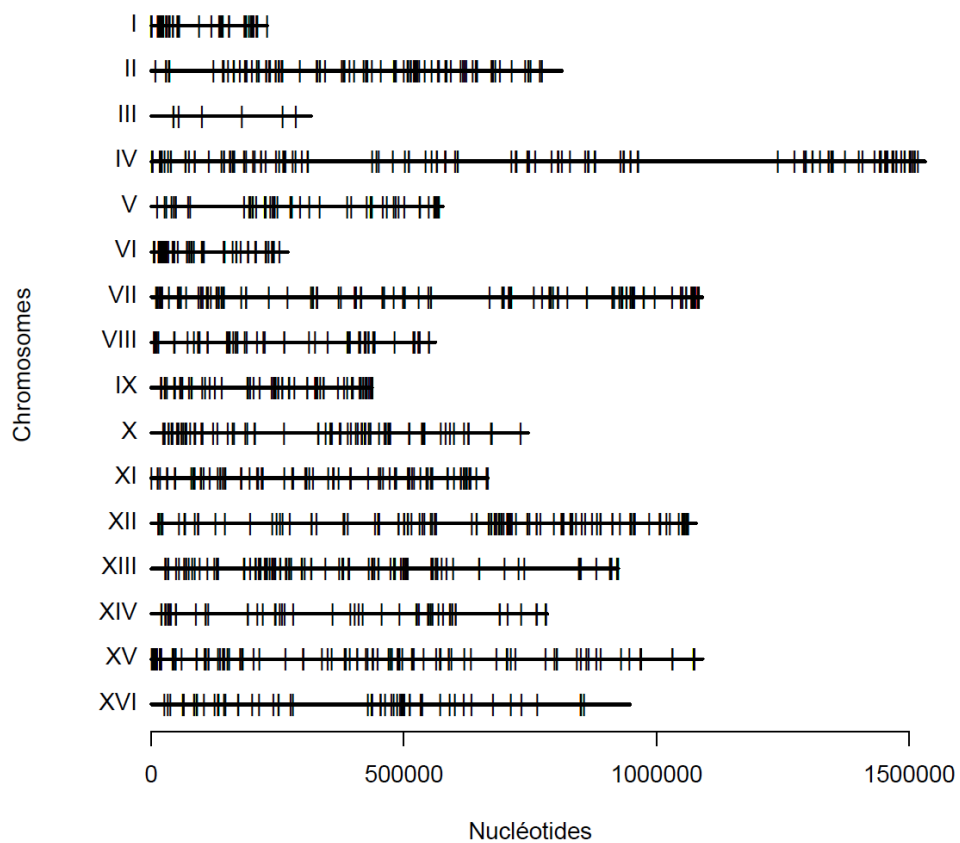

**Figure S5** Microarray-derived marker map. Each vertical tick represents one genetic marker.

Supplement: Supporting Information [file supp_1.4.263_FigureS5.pdf]
