# Supplementary material for: Deciphering the Molecular Basis of Wine Yeast Fermentation Traits Using a Combined Genetic and Genomic Approach
Source: G3 (Bethesda). 2011 Sep 1;1(4):263–81. doi: 10.1534/g3.111.000422 (PMC3276144; doi:10.1534/g3.111.000422)
Supplement: Supporting Information [file supp_1.4.263_FigureS6.pdf]

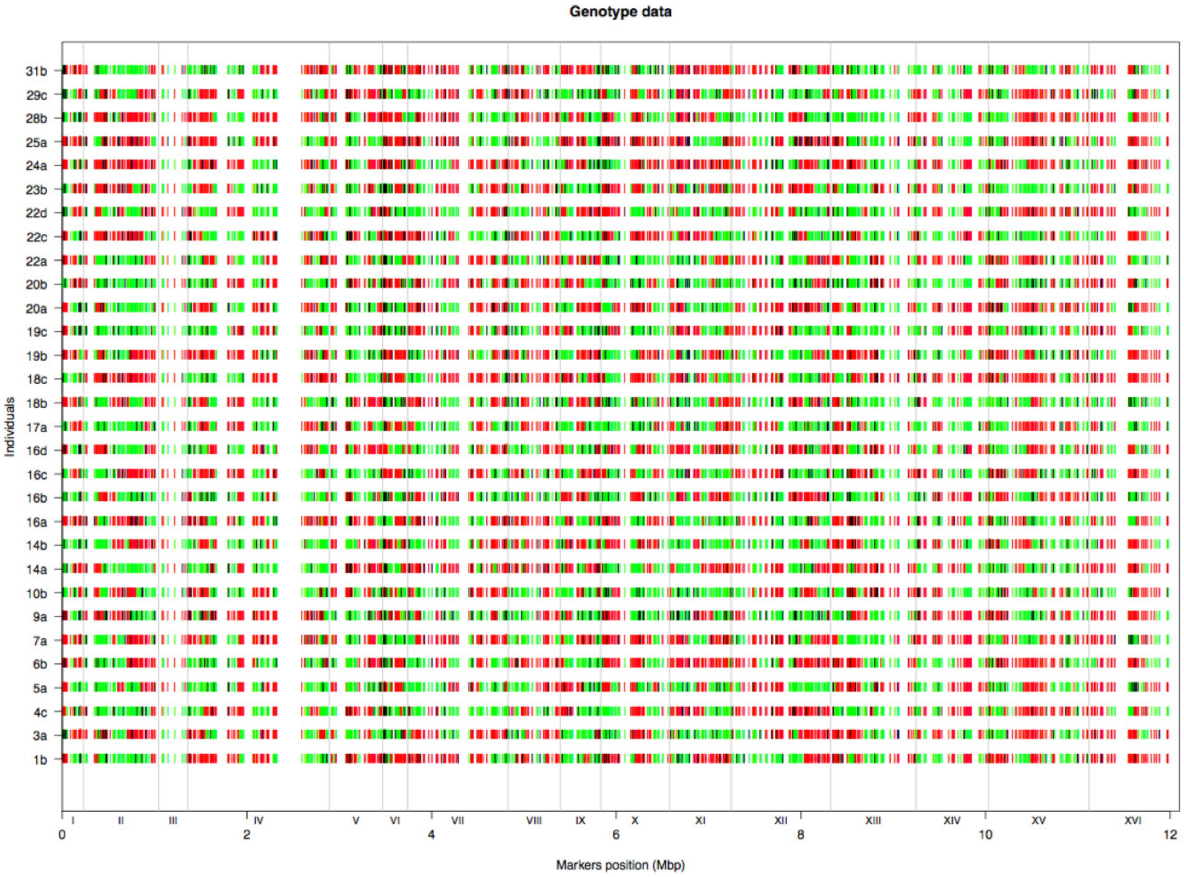

Figure S6 Markers distribution in the 30 segregants. Each marker position is displayed on the concatenated chromosomes with the colour indicating the parental origin (green 59A, red S288c). Ambiguous markers are indicated by a black bar.
