# Supplementary material for: Deciphering the Molecular Basis of Wine Yeast Fermentation Traits Using a Combined Genetic and Genomic Approach
Source: G3 (Bethesda). 2011 Sep 1;1(4):263–81. doi: 10.1534/g3.111.000422 (PMC3276144; doi:10.1534/g3.111.000422)
Supplement: Supporting Information [file supp_1.4.263_FigureS7.pdf]

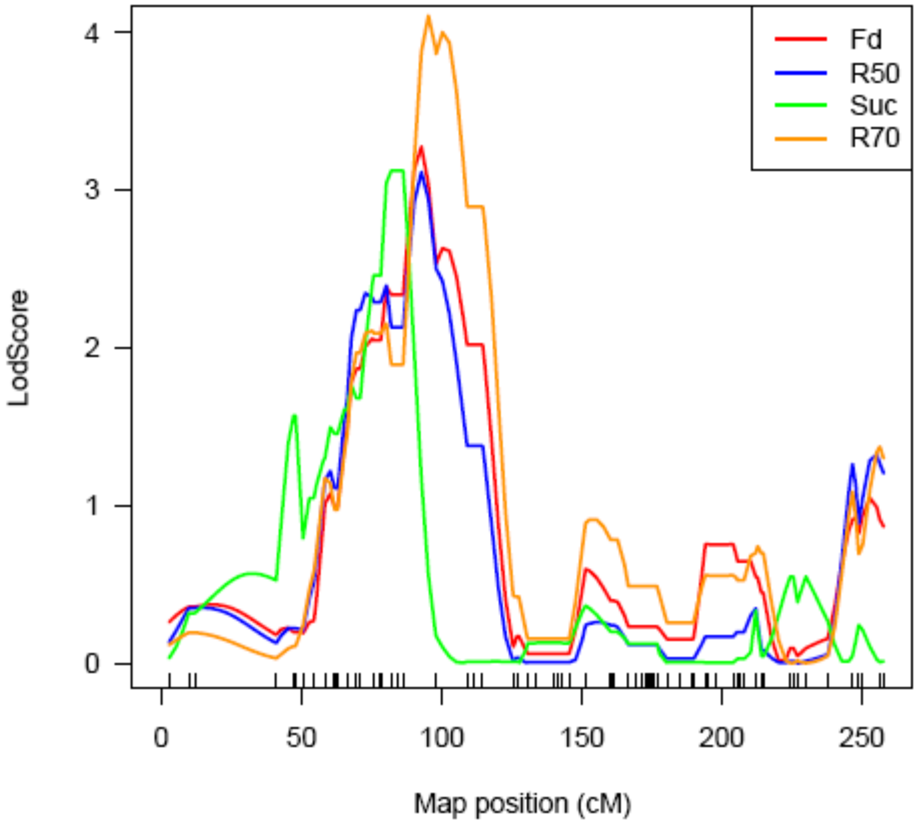

Figure S7 Overlapping of LOD peak values localised on chromosome II for four phenotypic traits. The x-axis represents the genomic marker localisation and the y-axis represents the LOD score values.
