# Supplementary material for: Deciphering the Molecular Basis of Wine Yeast Fermentation Traits Using a Combined Genetic and Genomic Approach
Source: G3 (Bethesda). 2011 Sep 1;1(4):263–81. doi: 10.1534/g3.111.000422 (PMC3276144; doi:10.1534/g3.111.000422)
Supplement: Supporting Information [file supp_1.4.263_FigureS8.pdf]

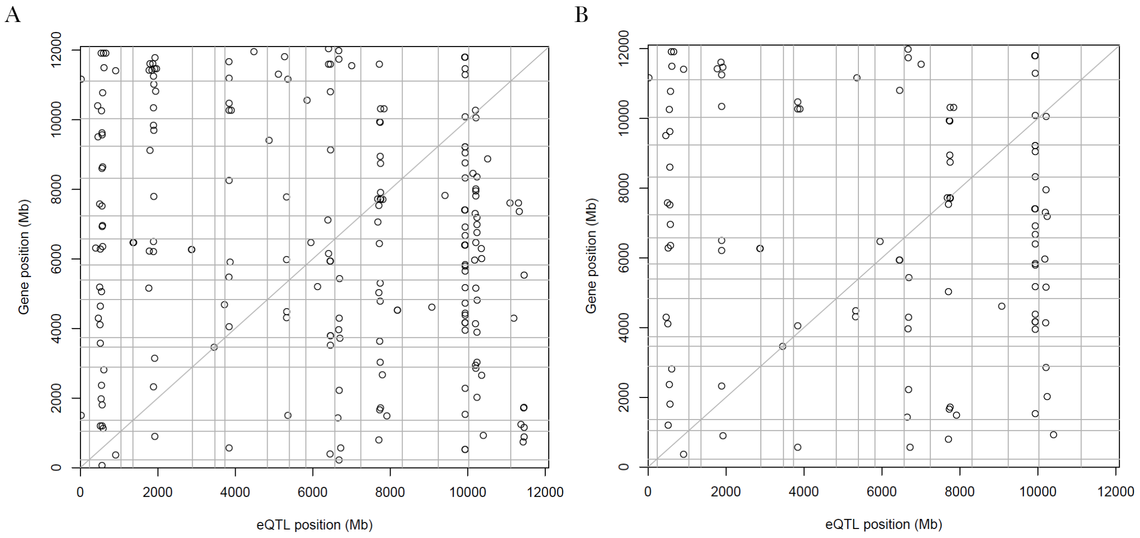

Figure S8 Genomic distribution of eQTL. Positions of detected eQTL are plotted against the position of the regulated gene. The figure displays eQTL with LOD score higher than 4.0 (A) and higher than 4.5 (B).
