# Supplementary material for: Deciphering the Molecular Basis of Wine Yeast Fermentation Traits Using a Combined Genetic and Genomic Approach
Source: G3 (Bethesda). 2011 Sep 1;1(4):263–81. doi: 10.1534/g3.111.000422 (PMC3276144; doi:10.1534/g3.111.000422)
Supplement: Supporting Information [file supp_1.4.263_FigureS9.pdf]

59 A	R	D	R	E	T
S. 288C	E N T G E	K K N I K	E F K D I	S V Q E Y	Y N K
RM11-1a	E N R G E	K K D I K	E F R D I	S V E E Y	Y T K
VIN13	E N R G E	K K D I K	E F R D I	S V E E Y	Y T K
YJM789	E N R G E	K K D I K	E F R D I	S V E E Y	Y T K
Y12	E N R G E	K K D I K	E F K D I	S V E E Y	Y T K
UWOPS87.2421	E N R G E	K K D I K	E F K D I	S V E E Y	Y T K
UWOPS05.217	E N R G E	K K D I K	E F K D I	S V E E Y	Y T K
S .bayanus	E N R G E	K K D V E	E F N D I	S V E E Y	Y T K
S. mikatae	E N R G E	K K D V R	E F N D I	S V E E Y	Y T K
S. paradoxus	E N R G E	K E D V R	E F N D I	S V E E Y	Y T K
	313	475	559	650	777

Figure S9 Alignment of the Abz1 amino acid modified in the strain 59A with the corresponding region seven other *Saccharomyces cerevisiae* strains and three other *Saccharomyces* species.
