# Supplementary material for: Deciphering the Molecular Basis of Wine Yeast Fermentation Traits Using a Combined Genetic and Genomic Approach
Source: G3 (Bethesda). 2011 Sep 1;1(4):263–81. doi: 10.1534/g3.111.000422 (PMC3276144; doi:10.1534/g3.111.000422)
Supplement: Supporting Information [file supp_1.4.263_TableS1.pdf]

Table S1 Parental strains and segregants fermentation characteristics

Strains	Rmax	R50	R70	Fd	Cp	Nass
S288C	1.63 (0.03)	1.04 (0.02)	0.81 (0.02)	128 (4.8)	194.9 (3.6)	234 (7)
59A	2.94 (0.05)	2.33 (0.17)	1.49 (0.08)	66 (0.7)	113.2 (3.5)	343 (0)
1b	2.96 (0.3)	2.54 (0.5)	2.07 (NA)	65 (4.2)	131.8 (8.3)	343 (NA)
3a	1.65 (0.13)	1.59 (0.08)	1.44 (0.12)	88 (4.9)	175 (0)	244 (NA)
4c	2.87 (0.33)	2.59 (0.34)	2.08 (0.22)	61 (2.5)	116.5 (26.9)	342 (NA)
5a	1.96 (0.3)	1.69 (0.27)	1.44 (0.18)	82 (4.9)	162.2 (11)	326 (NA)
6b	2.59 (0.42)	2.06 (0.35)	1.54 (0.13)	71 (4.6)	255 (59.4)	342 (NA)
7a	2.1 (0.21)	1.55 (0.25)	1.17 (0.15)	98 (12)	172.9 (2.7)	294 (NA)
9a	1.98 (0.26)	1.78 (0.1)	1.45 (0.03)	84 (4.9)	92.7 (11.7)	260 (NA)
10b	2.12 (0.93)	1.53 (0.56)	1.24 (0.04)	90 (16.3)	246.3 (43.7)	339 (NA)
14a	1.68 (0.04)	1.6 (0.04)	1.4 (0.01)	100 (0)	102.3 (18.8)	238 (NA)
14b	2.79 (0.42)	2.67 (0.37)	2.05 (0.15)	65 (7.1)	242.6 (11.5)	342 (NA)
16a	1.75 (0.15)	1.34 (0.16)	1 (0.08)	104 (6.4)	145.4 (7.3)	249 (NA)
16b	2.15 (0.13)	1.42 (0.07)	1.06 (0.07)	89 (5.7)	135.9 (4)	328 (NA)
16c	2.94 (0.42)	2.5 (0.22)	1.81 (0)	64 (3.9)	192.9 (6.2)	330 (NA)
16d	1.6 (0.21)	1.27 (0.26)	1.25 (0.21)	94 (10.5)	123.3 (9.6)	271 (NA)
17a	2.17 (0.47)	2.04 (0.4)	1.79 (0.32)	69 (11)	120.8 (6)	341 (NA)
18b	2.33 (0.21)	2.12 (0.25)	1.26 (NA)	85 (0.4)	193.5 (37.5)	330 (NA)
18c	1.79 (0.17)	1.3 (0.1)	1.12 (0)	102 (5.3)	124.1 (10.6)	309 (NA)
19b	2.46 (0.23)	1.62 (0.15)	1.07 (0.06)	88 (2.1)	154.3 (1.5)	335 (NA)
19c	1.64 (0.05)	1.56 (0.04)	1.32 (0.01)	96 (1.1)	69 (13.8)	287 (NA)
20a	1.74 (0.06)	1.7 (0.08)	1.5 (0.12)	88 (0.7)	90.6 (NA)	308 (NA)
20b	1.56 (0.08)	1.48 (0.08)	1.19 (NA)	98 (5.7)	95.8 (4.5)	251 (NA)
22a	2.71 (0.54)	2.42 (0.4)	1.99 (0.27)	63 (2.8)	161.1 (29.8)	342 (NA)
22c	1.47 (0.18)	1.5 (0.41)	0.67 (0.09)	149 (8.5)	132.2 (17.3)	258 (NA)
22d	2.94 (0.34)	2.38 (0.38)	1.77 (0.17)	65 (4.2)	NaN (NA)	343 (NA)
23b	2.07 (0.25)	1.29 (0.3)	0.96 (0.14)	100 (5.7)	150.2 (12.9)	326 (NA)
24a	2.48 (0.48)	1.61 (0.69)	1.05 (0.15)	90 (9)	319.9 (37.6)	341 (NA)
25a	1.53 (0.18)	1.21 (0.09)	0.95 (0.18)	114 (23.3)	191.6 (NA)	241 (NA)
28b	2.34 (0.68)	2.31 (0.69)	1.99 (0.5)	80 (12)	148.6 (12.8)	316 (NA)
29c	1.21 (0.08)	0.86 (0.08)	0.7 (0.03)	144 (6.4)	134.4 (8.9)	276 (NA)
31b	2.15 (0.31)	2.1 (0.3)	1.85 (0.28)	80 (3.5)	108.8 (18)	328 (NA)

Standard deviation for each trait is indicated between parentheses.
