# Supplementary material for: Deciphering the Molecular Basis of Wine Yeast Fermentation Traits Using a Combined Genetic and Genomic Approach
Source: G3 (Bethesda). 2011 Sep 1;1(4):263–81. doi: 10.1534/g3.111.000422 (PMC3276144; doi:10.1534/g3.111.000422)
Supplement: Supporting Information [file supp_1.4.263_TableS2.pdf]

Table S2 Parental strains and segregants metabolites production characteristics

Strains	Suc	Gly	Ace	Pyr
S288C	0.23 (0.01)	5.55 (0.12)	1.12 (0.04)	0.06 (0.02)
59A	0.26 (0.03)	6.5 (0.06)	0.65 (0.06)	0.1 (0.01)
1b	0.3 (0.09)	5.31 (0.4)	0.45 (0.04)	0.13 (0.02)
3a	0.36 (0.07)	6.49 (0.56)	1.35 (0.11)	0.21 (0.05)
4c	0.26 (0.01)	5 (0.24)	0.28 (0.08)	0.21 (0)
5a	0.28 (0)	5.61 (0.21)	1.01 (0.13)	0.27 (0.05)
6b	0.26 (0.07)	5.56 (0.45)	0.85 (0.17)	0.22 (0.06)
7a	0.26 (0.02)	4.88 (0.44)	1.11 (0.03)	0.13 (0.02)
9a	0.25 (0.02)	5.19 (0.42)	0.99 (0.01)	0.25 (0.09)
10b	0.34 (0.1)	5.8 (0.3)	0.88 (0.2)	0.12 (0.07)
14a	0.31 (NA)	7.01 (NA)	1.33 (NA)	0.31 (NA)
14b	0.26 (0)	4.66 (0.4)	0.38 (0.04)	0.2 (0.01)
16a	0.26 (0.01)	5.54 (0.31)	0.97 (0.09)	0.14 (0.06)
16b	0.29 (0.05)	4.84 (0.26)	0.81 (0.14)	0.11 (0.06)
16c	0.3 (0.07)	5.41 (0.32)	0.72 (0.14)	0.12 (0.02)
16d	0.42 (0.01)	6.66 (0.44)	1.46 (0.21)	0.18 (0.01)
17a	0.31 (0.04)	6.88 (0.82)	0.93 (0.02)	0.25 (0)
18b	0.3 (0.03)	5.89 (0.47)	0.53 (0.1)	0.12 (0.02)
18c	0.31 (0)	5.23 (0.3)	1.23 (0.12)	0.14 (0.02)
19b	0.26 (0.06)	5.13 (0.43)	0.58 (0.11)	0.08 (0.01)
19c	0.3 (0.03)	6.7 (0.38)	1.23 (0.14)	0.26 (0.06)
20a	0.34 (0.03)	5.95 (0.33)	0.8 (0.15)	0.21 (0.01)
20b	0.34 (0.06)	5.78 (0.43)	1.25 (0.01)	0.16 (0.08)
22a	0.28 (0.08)	5.49 (0.32)	0.73 (0.16)	0.19 (0.05)
22c	0.28 (0)	4.48 (0.37)	0.99 (0.07)	0.09 (0)
22d	0.31 (0.1)	5.5 (0.32)	0.76 (0.19)	0.25 (0.05)
23b	0.45 (0.01)	5.81 (0.4)	1.03 (0.11)	0.12 (0.01)
24a	0.36 (0.03)	4.93 (0.26)	0.61 (0.2)	0.07 (0)
25a	0.33 (0.03)	6.47 (0.76)	1.25 (0.12)	0.21 (0.09)
28b	0.45 (0.04)	5.48 (0.23)	0.33 (0.01)	0.28 (0.13)
29c	0.35 (0.03)	5.57 (0.44)	0.95 (0.1)	0.13 (0.01)
31b	0.24 (0.03)	5.32 (0.13)	0.8 (0.04)	0.37 (0.07)

Standard deviation for each trait is indicated between parentheses.
