# Supplementary material for: Deciphering the Molecular Basis of Wine Yeast Fermentation Traits Using a Combined Genetic and Genomic Approach
Source: G3 (Bethesda). 2011 Sep 1;1(4):263–81. doi: 10.1534/g3.111.000422 (PMC3276144; doi:10.1534/g3.111.000422)
Supplement: Supporting Information [file supp_1.4.263_TableS3.pdf]

**Table S3 Genes showing strong differential expression between the two parental strains. (A) Genes overexpressed in 59A. (B) Genes overexpressed in S288c.**

**A**

| Name | logFC | Ratio | Adj PVal | Function |
|---|---|---|---|---|
| PAU5 | 3.256 | 9.555 | 8.56E-16 | Part of 23-member seripauperin multigene family encoded mainly in subtelomeric regions |
| PAU4 | 3.242 | 9.458 | 9.26E-15 | Part of 23-member seripauperin multigene family encoded mainly in subtelomeric regions |
| PAU12 | 2.83 | 7.112 | 1.97E-15 | Hypothetical protein |
| PAU20 | 2.827 | 7.094 | 3.61E-15 | Hypothetical protein |
| PAU6 | 2.483 | 5.592 | 1.43E-13 | Part of 23-member seripauperin multigene family encoded mainly in subtelomeric regions |
| PAU1 | 2.412 | 5.322 | 1.78E-14 | Part of 23-member seripauperin multigene family encoded mainly in subtelomeric regions |
| PAU13 | 2.324 | 5.007 | 8.02E-13 | Putative protein of unknown function |
| INO1 | 2.287 | 4.88 | 1.05E-12 | Inositol 1-phosphate synthase |
| PAU10 | 2.281 | 4.861 | 7.24E-14 | Hypothetical protein |
| PAU14 | 2.239 | 4.721 | 7.27E-14 | Hypothetical protein |
| PAU11 | 2.214 | 4.64 | 7.88E-13 | Putative protein of unknown function |
| PAU8 | 2.133 | 4.385 | 7.85E-12 | Hypothetical protein |
| PAU7 | 2.038 | 4.107 | 3.78E-14 | Part of 23-member seripauperin multigene family |
| PAU18 | 1.973 | 3.926 | 1.96E-12 | Hypothetical protein |
| HXK1 | 1.877 | 3.674 | 2.70E-10 | Hexokinase isoenzyme 1 |
| PAU19 | 1.85 | 3.606 | 3.87E-13 | Hypothetical protein |
| HUG1 | 1.752 | 3.368 | 1.51E-11 | Protein involved in the Mec1p-mediated checkpoint pathway that responds to DNA damage or replication arrest |
| TRK2 | 1.642 | 3.122 | 2.47E-11 | Component of the Trk1p-Trk2p potassium transport system |
| YGR126W | 1.641 | 3.119 | 3.61E-11 | Putative protein of unknown function |
| YNL324W | 1.638 | 3.112 | 2.74E-12 | Dubious ORF unlikely to encode a functional protein |
| YDR102C | 1.589 | 3.008 | 4.15E-12 | Dubious open reading frame |
| YPR126C | 1.558 | 2.945 | 2.35E-14 | Dubious open reading frame unlikely to encode a functional protein |
| DAN3 | 1.549 | 2.925 | 5.89E-10 | Cell wall mannoprotein with similarity to Tir1p |
| DDR48 | 1.544 | 2.917 | 1.37E-07 | DNA damage-responsive protein |
| DAN2 | 1.513 | 2.855 | 1.23E-12 | Cell wall mannoprotein with similarity to Tir1p |
| GPH1 | 1.437 | 2.707 | 1.05E-12 | Non-essential glycogen phosphorylase required for the mobilization of glycogen |
| MFA2 | 1.413 | 2.664 | 4.15E-09 | Mating pheromone a-factor |
| YDL085C-A | 1.402 | 2.643 | 8.24E-09 | Putative protein of unknown function |
| YPR050C | 1.373 | 2.59 | 1.28E-12 | Dubious open reading frame unlikely to encode a protein |
| YDR355C | 1.334 | 2.521 | 9.76E-12 | Dubious: Dubious open reading frame unlikely to encode a protein |
| YIL067C | 1.326 | 2.507 | 5.74E-12 | Uncharacterized protein of unknown function |
| YCL020W | 1.302 | 2.466 | 2.57E-09 | Retrotransposon TYA Gag gene co-transcribed with TYB Pol |
| SOL2 | 1.301 | 2.464 | 9.67E-11 | Protein with a possible role in tRNA export |
| JSN1 | 1.272 | 2.415 | 9.71E-14 | Member of the Puf family of RNA-binding proteins |
| GPM1 | 1.271 | 2.412 | 7.16E-10 | Tetrameric phosphoglycerate mutase |
| MSG5 | 1.256 | 2.389 | 4.09E-10 | Dual-specificity protein phosphatase required for maintenance of a low level of signaling through the cell integrity pathway |
| DBP5 | 1.248 | 2.375 | 3.52E-10 | Cytoplasmic ATP-dependent RNA helicase of the DEAD-box family involved in mRNA export from the nucleus |
| YBR099C | 1.246 | 2.372 | 4.78E-11 | Dubious open reading frame unlikely to encode a protein |
| SDS24 | 1.223 | 2.334 | 1.77E-10 | One of two S |
| HRT1 | 1.221 | 2.33 | 1.20E-11 | RING finger containing subunit of Skp1-Cullin-F-box ubiquitin protein ligases (SCF) |
| PAU17 | 1.218 | 2.326 | 2.77E-09 | Putative protein of unknown function |
| PEX11 | 1.207 | 2.309 | 8.92E-11 | Peroxisomal membrane protein required for peroxisome proliferation and medium-chain fatty acid oxidation |

| | | | | |
|---|---|---|---|---|
| MSH6 | 1.206 | 2.307 | 4.89E-09 | Protein required for mismatch repair in mitosis and meiosis |
| FLO5 | 1.205 | 2.306 | 1.71E-13 | Lectin-like cell wall protein (flocculin) involved in flocculation |
| YFL052W | 1.205 | 2.306 | 1.93E-13 | Putative zinc cluster protein that contains a DNA binding domain |
| YKR045C | 1.202 | 2.3 | 2.57E-10 | Putative protein of unknown function |
| YJL127W-A | 1.201 | 2.298 | 1.22E-08 | Dubious open reading frame unlikely to encode a protein |
| DAN1 | 1.2 | 2.297 | 3.92E-10 | Cell wall mannoprotein with similarity to Tir1p |
| ENP1 | 1.189 | 2.28 | 2.20E-08 | Protein associated with U3 and U14 snoRNAs |
| YHR049C-A | 1.188 | 2.279 | 7.03E-11 | Dubious open reading frame unlikely to encode a functional protein |

**B**

| Name | logFC | Ratio | Adj PVal | Function |
|---|---|---|---|---|
| PLC1 | 4.693 | 25.864 | 4.07E-19 | Phospholipase C |
| YNL284C-A* | 4.252 | 19.047 | 1.89E-19 | Retrotransposon TYA Gag gene co-transcribed with TYB Pol |
| AI2* | 4.235 | 18.833 | 3.92E-18 | Reverse transcriptase required for splicing of the COX1 pre-mRNA |
| RTC4 | 3.439 | 10.844 | 3.68E-18 | Putative protein of unknown function |
| CDC5 | 2.857 | 7.246 | 2.82E-17 | Polo-like kinase with similarity to Xenopus Plx1 and S |
| ARH1 | 2.77 | 6.823 | 1.55E-17 | Oxidoreductase of the mitochondrial inner membrane |
| YGR051C | 2.53 | 5.776 | 2.24E-17 | Dubious open reading frame unlikely to encode a protein |
| YER084W | 2.49 | 5.617 | 4.91E-16 | Dubious open reading frame unlikely to encode a protein |
| YNL285W | 2.459 | 5.498 | 1.12E-17 | Dubious open reading frame unlikely to encode a functional protein |
| ELG1 | 2.437 | 5.413 | 1.57E-14 | Protein required for S phase progression and telomere homeostasis |
| ATE1 | 2.41 | 5.316 | 1.89E-19 | Arginyl-tRNA-protein transferase |
| YER138W-A | 2.379 | 5.201 | 5.74E-12 | Putative protein of unknown function |
| SCH9 | 2.365 | 5.152 | 3.30E-16 | Protein kinase involved in transcriptional activation of osmostress-responsive genes |
| YBL107W-A | 2.234 | 4.705 | 6.28E-14 | Dubious open reading frame unlikely to encode a protein |
| RSA1 | 2.204 | 4.609 | 5.55E-18 | Protein involved in the assembly of 60S ribosomal subunits |
| THI80 | 2.157 | 4.459 | 1.55E-16 | Thiamine pyrophosphokinase |
| HSP30 | 2.121 | 4.349 | 3.76E-08 | Hydrophobic plasma membrane localized |
| YBL111C | 2.068 | 4.192 | 9.79E-18 | Helicase-like protein encoded within the telomeric Y element |
| YHR218W | 2.018 | 4.051 | 2.21E-16 | Helicase-like protein encoded within the telomeric Y element |
| YIR014W | 1.93 | 3.809 | 2.68E-15 | Putative protein of unknown function |
| GIN4 | 1.894 | 3.717 | 1.51E-17 | Protein kinase involved in bud growth and assembly of the septin ring |
| HSP12 | 1.885 | 3.695 | 2.94E-14 | Plasma membrane localized protein that protects membranes from desiccation |
| PBP1 | 1.857 | 3.623 | 1.05E-14 | Protein interacting with poly(A)-binding protein Pab1p |
| YHL005C | 1.842 | 3.584 | 6.15E-17 | Dubious open reading frame unlikely to encode a protein |
| AUA1 | 1.801 | 3.485 | 8.96E-09 | Protein required for the negative regulation by ammonia of Gap1p |
| TPO3 | 1.8 | 3.481 | 9.65E-14 | Polyamine transport protein specific for spermine |
| RPL2B | 1.767 | 3.402 | 2.51E-14 | Protein component of the large (60S) ribosomal subunit |
| YER053C-A | 1.745 | 3.352 | 1.89E-06 | Putative protein of unknown function |
| MCM22 | 1.737 | 3.334 | 6.76E-15 | Protein involved in minichromosome maintenance |
| ASP3-1 | 1.734 | 3.326 | 9.66E-15 | Cell-wall L-asparaginase II |
| SPS100 | 1.712 | 3.276 | 1.94E-11 | Protein required for spore wall maturation |
| ASP3-3 | 1.704 | 3.257 | 4.88E-14 | Cell-wall L-asparaginase II |
| NPY1 | 1.669 | 3.18 | 5.89E-15 | NADH diphosphatase (pyrophosphatase) |
| HST1 | 1.665 | 3.172 | 3.56E-15 | NAD(+)-dependent histone deacetylase |
| YLR281C | 1.665 | 3.17 | 1.01E-16 | Putative protein of unknown function |
| SPR3 | 1.644 | 3.125 | 3.61E-15 | Sporulation-specific homolog of the yeast CDC3/10/11/12 family of bud neck microfilament genes |
| ASP3-2 | 1.628 | 3.09 | 1.70E-15 | Cell-wall L-asparaginase II |
| YNL260C | 1.614 | 3.06 | 1.72E-15 | Putative protein of unknown function with similarity to a human protein overexpressed in oral cancers |
| KAR9 | 1.581 | 2.991 | 8.55E-10 | Karyogamy protein required for correct positioning of the mitotic spindle and for orienting cytoplasmic microtubules |
| VIP1 | 1.577 | 2.984 | 8.36E-14 | Inositol hexakisphosphate (IP6) and inositol heptakisphosphate (IP7) kinase |

| | | | | |
|---|---|---|---|---|
| DOT1 | 1.572 | 2.973 | 1.32E-13 | Nucleosomal histone H3-Lys79 methylase |
| AI3 | 1.557 | 2.942 | 1.99E-13 | Endonuclease I-SceIII |
| MRPL13 | 1.54 | 2.909 | 6.20E-13 | Mitochondrial ribosomal protein of the large subunit |
| YLR280C | 1.54 | 2.909 | 5.89E-15 | Dubious open reading frame unlikely to encode a functional protein |
| ASP3-4 | 1.536 | 2.899 | 2.40E-15 | Cell-wall L-asparaginase II |
| ITC1 | 1.519 | 2.865 | 6.51E-14 | Component of the ATP-dependent Isw2p-Itc1p chromatin remodeling complex |
| YIL089W | 1.511 | 2.851 | 6.00E-13 | Putative protein of unknown function |
| RPA49 | 1.498 | 2.824 | 7.21E-06 | RNA polymerase I subunit A49 |
| HXT5 | -1.477 | 2.783 | 3.80 E-15 | Hexose transporter |
| THI72 | -1,477 | 2.783 | 1.71 E-11 | Transporter of thiamine or related compound |

For each gene the $\log_2$ of fold change, the ratio of expression, the adjusted p-value and the function are indicated.
These are Ty1 genes, only one member of each was considered since they cross hybridize.
