# Supplementary material for: Deciphering the Molecular Basis of Wine Yeast Fermentation Traits Using a Combined Genetic and Genomic Approach
Source: G3 (Bethesda). 2011 Sep 1;1(4):263–81. doi: 10.1534/g3.111.000422 (PMC3276144; doi:10.1534/g3.111.000422)
Supplement: Supporting Information [file supp_1.4.263_TableS4.pdf]

Table S4 Genes with eQTL in the 7 hotspots. Hotspot lod > 3.5

Hotspot	Gene				
**Chr II**	YAL044W-A	SSZ1	YMR031C	PMP3	MNE1
**87.8 - 125.3 cM**	FLC2	SPS100	NRK1	HSP12	ISA2
**263400 – 3759000 bp**	TDP1	CYR1	RPL42A	FMP37	PMT6
	HSP30	HXT8	COQ10	YGR079W	ECM12
	NTH1	SPC1	GSH2	CYS4	GCN3
	YDR053W	OSM1	DDR2	PTM1	EMP46
	TVP23	GPX1	MCT1	YKL165C-A	YMR010W
**Chr IV**	SFT2	STR2	YOR352W	YJL028W	SWR1
**161 – 188.2 cM**	DAD1	PNP1	SMA1	ISY1	RHO2
**483000 – 564600 bp**	YDR249C	YNL019C	ELP4	IZH2	FMP40
	YPL264C				
**Chr XI**	YAR069C	TIM21	YLR104W	YFR016C	SEC4
**29.2 – 45.5**	MRPL11	THI4	SIP3	FRS1	
**87600 – 136500 bp**	UME6	POT1	CSR2	ORC4	
**Chr XII**	CWH43	RPL43B	CTF18	SHE10	YPL034W
**147.7 – 171.6 cM**	MCH1	ASP3-1	SSO2	YOR1	GSM1
**443100 – 514800 bp**	YDL086W	ASP3-2	NGL2	IKI1	
	YEL008W	ASP3-3	HUB1	VPS63	
	IOC3	ASP3-4	MRPS12	YUH1	
**Chr XIV**	CDC15	HAM1	MTF1	VOA1	DDC1
**211.6 - 244 cM**	OLA1	MOG1	YMR262W	ENP2	JID1
**634800 – 732000 bp**	SAS10	YKL050C	DIA1	YGR251W	YIL055C
	DHH1	MTR2	ATG4	YRF1-3	RPS24B
	PCF11	SPA2	RRP40	LSM12	MDE1
	PNC1	YLR012C	YOR021C	YML131W	YJL225C
	NSA1	GAT3	RPL3	YMR086W	
	PEX14	RFX1	YOR289W	MRS1	
	PIL1	YLR243W	YPL107W	KAR2	
**Chr XV**	PGS1	CDC12	CHS5	SWC4	YOL047C
**51.4 - 71.4 cM**	ESF1	KRE27	RSC2	YGR265W	YOR050C
**154200 – 214200 bp**	MUS81	SNX4	ATG17	YHR003C	GCY1
	YDR532C	EXO70	OGG1	YLR257W	DSS4
	AFG1	NNF1	ALO1	MEC3	FRE1
	MST27	MLP1	ILV2	SFH1	VPS75
	HAP2				
**Chr XVI**	ATG12	MRP10	YEL076C	IDP3	
**96 – 126 cM**	YCP4	MCH1	ROK1	YEL008W	
**288000 – 378000 bp**	BUD23	CHL4	HIS5	YCR101C	

Genes which have a LOD score higher than 3.5 in the hotspot are displayed.
